# Insight into the AP2/ERF transcription factor superfamily in sesame and expression profiling of DREB subfamily under drought stress

**DOI:** 10.1186/s12870-016-0859-4

**Published:** 2016-07-30

**Authors:** Komivi Dossa, Xin Wei, Donghua Li, Daniel Fonceka, Yanxin Zhang, Linhai Wang, Jingyin Yu, Liao Boshou, Diaga Diouf, Ndiaga Cissé, Xiurong Zhang

**Affiliations:** 1 Oil Crops Research Institute of the Chinese Academy of Agricultural Sciences, Key Laboratory of Biology and Genetic Improvement of Oil Crops, Ministry of Agriculture, No.2 Xudong 2nd Road, 430062 Wuhan, Hubei China; 2 Centre d’Etudes Régional pour l’Amélioration de l’Adaptation à la Sécheresse (CERAAS), BP 3320 Route de Khombole, Thiès, Sénégal; 3 Laboratoire Campus de Biotechnologies Végétales, Département de Biologie Végétale, Faculté des Sciences et Techniques, Université Cheikh Anta Diop, BP 5005 Dakar-Fann, Code postal 107000 Dakar, Sénégal; 4 CIRAD, UMR AGAP, F-34398 Montpellier, France

**Keywords:** *Sesamum indicum*, AP2/ERF, Transcription factors, Gene expression, Drought stress

## Abstract

**Background:**

Sesame is an important oilseed crop mainly grown in inclement areas with high temperatures and frequent drought. Thus, drought constitutes one of the major constraints of its production. The AP2/ERF is a large family of transcription factors known to play significant roles in various plant processes including biotic and abiotic stress responses. Despite their importance, little is known about sesame AP2/ERF genes. This constitutes a limitation for drought-tolerance candidate genes discovery and breeding for tolerance to water deficit.

**Results:**

One hundred thirty-two AP2/ERF genes were identified in the sesame genome. Based on the number of domains, conserved motifs, genes structure and phylogenetic analysis including 5 relatives species, they were classified into 24 AP2, 41 DREB, 61 ERF, 4 RAV and 2 Soloist. The number of sesame AP2/ERF genes was relatively few compared to that of other relatives, probably due to gene loss in ERF and DREB subfamilies during evolutionary process. In general, the AP2/ERF genes were expressed differently in different tissues but exhibited the highest expression levels in the root. Mostly all DREB genes were responsive to drought stress. Regulation by drought is not specific to one DREB group but depends on the genes and the group A6 and A1 appeared to be more actively expressed to cope with drought.

**Conclusions:**

This study provides insights into the classification, evolution and basic functional analysis of AP2/ERF genes in sesame which revealed their putative involvement in multiple tissue-/developmental stages. Out of 20 genes which were significantly up- /down-regulated under drought stress, the gene AP2si16 may be considered as potential candidate gene for further functional validation as well for utilization in sesame improvement programs for drought stress tolerance.

**Electronic supplementary material:**

The online version of this article (doi:10.1186/s12870-016-0859-4) contains supplementary material, which is available to authorized users.

## Background

Sesame (*Sesamum indicum* L., 2*n* = 2x = 26) is an oil crop that contributes to the daily oil and protein requirements of almost half of the world’s population [1]. It is a high nutritive value crop thanks to its oil quality and quantity ranging from 40 to 62.7 % [2]. It was reported that sesame contain much compounds that benefit to human health, including antioxidant, antiaging, antihypertensive, anticancer, cholesterol lowering and antimutagenic properties [3–5]. The global demand for vegetable oils is growing and estimated to reach 240 million tons by 2050 [6]. Sesame is therefore a productive plant which may greatly contribute to meet this demand. However to reach this objective, it is important to alleviate the different constraints that impair the crop productivity. Water deficit or drought considered to be one of the greatest abiotic factors that limit global food production [7] is significantly affecting 64 % of the global land area [8]. Drought is one of the major constraints of sesame production especially because it is mainly grown in arid and semi-arid regions where the occurrence of drought is frequent [9]. The crop is highly sensitive to drought during its vegetative stage and its yield is adversely affected by water scarcity [10–12]. In addition, the negative effect of drought stress on the sesame oil quality has been reported [13–15].

Osmotic stresses including drought induce a cascade of molecular responses in plants. Many stress-responsive genes are expressed differentially to adapt to unfavorable environmental conditions. Induction of stress-related genes occurs mainly at the transcriptional level. The modification of the temporal and spatial expression patterns of the specific stress-related genes is an important part of the plant stress response [16]. Transcription factors (TFs) act as regulatory proteins by regulating in a synchronized manner a set of targeted genes under their control and consequently enhance the stress tolerance of the plant. Among these transcription factors, the AP2/ERF superfamily constitutes one of the biggest gene families, which contains a typical AP2 DNA-binding domain and is widely present in plants [17]. The AP2/ERF superfamily is involved in response to drought, to high-salt content, to temperature change, to disease resistance, in flowering control pathway and has been analyzed by a combination of genetic and molecular approaches [18].

According to the classification of Sakuma et al. (2002) [19] and later on adopted by several authors [20–23], AP2/ERF superfamily includes five subfamilies: (1) AP2 (APETALA2), (2) DREB (dehydration-responsive-element-binding), (3) RAV (related to ABI3/VP), (4) ERF (ethylene-responsive-element-binding-factor), and (5) other proteins (Soloist), based on the number of AP2/ERF domains and the presence of other DNA binding domains. While the AP2 family contains two repeated AP2/ERF domains, the ERF and DREB subfamilies contain a single AP2/ERF domain [24]. The RAV family contains a single AP2/ERF domain and a specific B3 motif [25]. The extensive genomic studies of the AP2/ERF superfamily in *Arabidopsis* [19, 26], poplar [20], soybean [27], rice [28], grape [20, 23], cucumber [29], hevea [30], castor bean [31], Chinese cabbage [32], foxtail millet [33], *Salix arbutifolia* [22] and *Eucalyptus grandis* [23] have provided a better understanding of these TFs. This gene family is highly conserved in plant species although number of gene, functional groups, and gene function could differ according to the species, as a result of independent and different evolution processes. There are many evidences of implication of AP2/ERF genes especially DREBs in drought stress responses in crops [34]. In *Arabidopsis*, DREB2A and DREB2B are reported to be induced by dehydration [19, 35–37]. The soybean GmDREB2 protein has also been reported to promote the expression of downstream genes to enhance drought tolerance in transgenic *Arabidopsis* [38].

The lack of gene resources associated with drought tolerance hinders genetic improvement in sesame [39]. The recent advances on the sesame genome sequence and the identification of its complement of 27,148 have brought sesame genome research into the functional genomics age [40]. This makes possible genome-wide analysis to find out valuable genes linked to important traits such as drought and to support sesame-breeding programs.

Since little is known about the important AP2/ERF superfamily in sesame, here, we described these TFs and analysed the potential role of DREB subfamily in responses to drought stress. This study will pave the way for the comprehensive analysis and the understanding of the biological roles of AP2/ERF genes in sesame towards the improvement of drought stress tolerance.

## Results

### Identification and chromosomal location of the AP2/ERF gene superfamily

A total of 132 AP2/ERF genes were confirmed in sesame with complete AP2-type DNA-binding domains ranging from 273 to 5837 bp in length (Table 1; Additional file 1). These genes represent about 0.55 % of the total number of genes in sesame. Based on the nature and the number of DNA-binding domains, they were further divided into four major families namely AP2, ERF, DREB and RAV. Twenty genes were predicted to encode proteins containing double-repeated AP2/ERF domains (AP2 family). Four genes were predicted to encode single AP2/ERF domain, together with one B3 domain (RAV family). One hundred and two genes were predicted to encode proteins containing single AP2/ERF domain (ERF family) including 61 genes assigned to the ERF subfamily and 41 genes assigned to the DREB subfamily. Out of the remaining six genes, four genes (*AP2si132*, *AP2si117*, *AP2si58* and *AP2si131*) encoded single AP2/ERF domain distinct from those of the members of the ERF family but were more closely related to those of the AP2 family members. Thus these four genes were then assigned to the AP2 family. Finally, the last two genes (*AP2si91* and *AP2si96*) also contained single AP2 domain which showed a low similarity with the AP2 and ERF families. It was found that they have a high similarity with the amino acid sequence of the *Arabidopsis* gene “*At4g13040*” classified as “Soloist”. Therefore, these genes were designated as “Soloist”.

**Table 1:** Classification of the AP2/ERF superfamily in sesame

Classification	Group	Number
AP2 family	Double AP2/ERF domain	20
	Single AP2/ERF	4
	domain	
ERF family	DREB subfamily	41
	A1	9
	A2	5
	A3	1
	A4	10
	A5	6
	A6	10
	ERF subfamily	61
	B1	5
	B2	4
	B3	22
	B4	6
	B5	19
	B6	5
RAV family		4
Soloist		2
	Total	132

The names of families, subfamilies and groups were previously reported by Sakuma et al. [19]

**Fig. 1:**
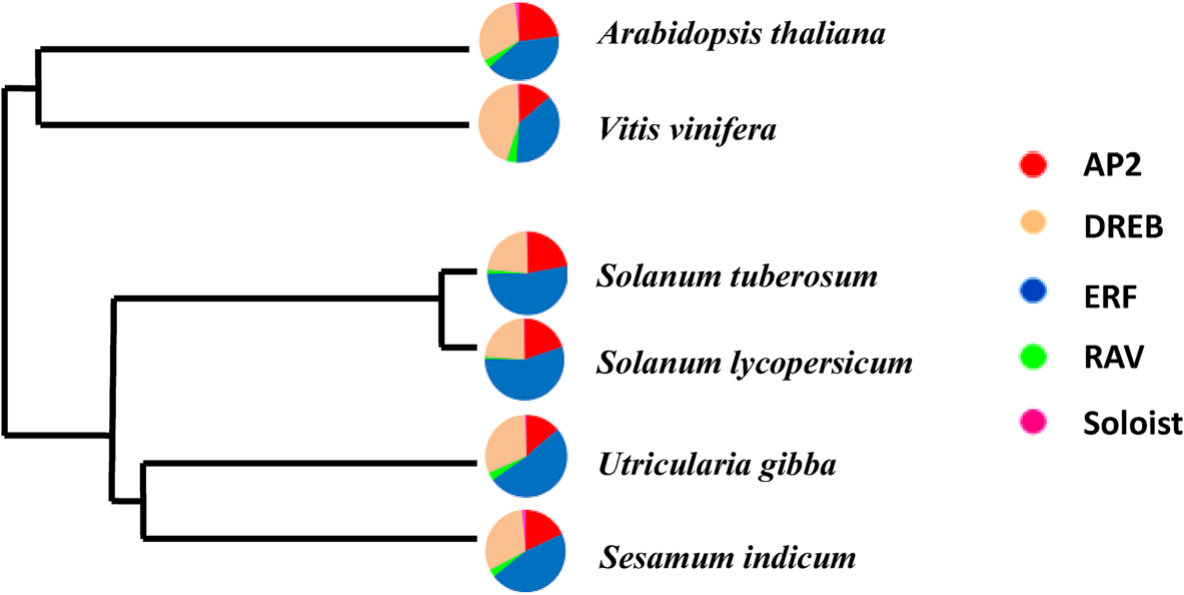
Phylogeny of the six species and repartition of the AP2/ERF families

Cumulatively, the number of AP2/ERF genes in sesame is slightly lower than the five relative species analyzed: *Arabidopsis* (147) [19], grape (149) [24], *U. gibba* (152) [41], tomato (167) [42] and potato (246) [43]. As described for these species relatives, ERF and AP2 families were also overrepresented in the sesame genome. *Arabidopsis* and sesame have three and two “Soloist” genes respectively while the other four species have only one “Soloist” gene in their genomes. The Fig. 1 summarizes the AP2/ERF superfamily members detected in grape, tomato, potato, *Arabidopsis*, *Utricularia gibba* and sesame.

**Fig. 2:**
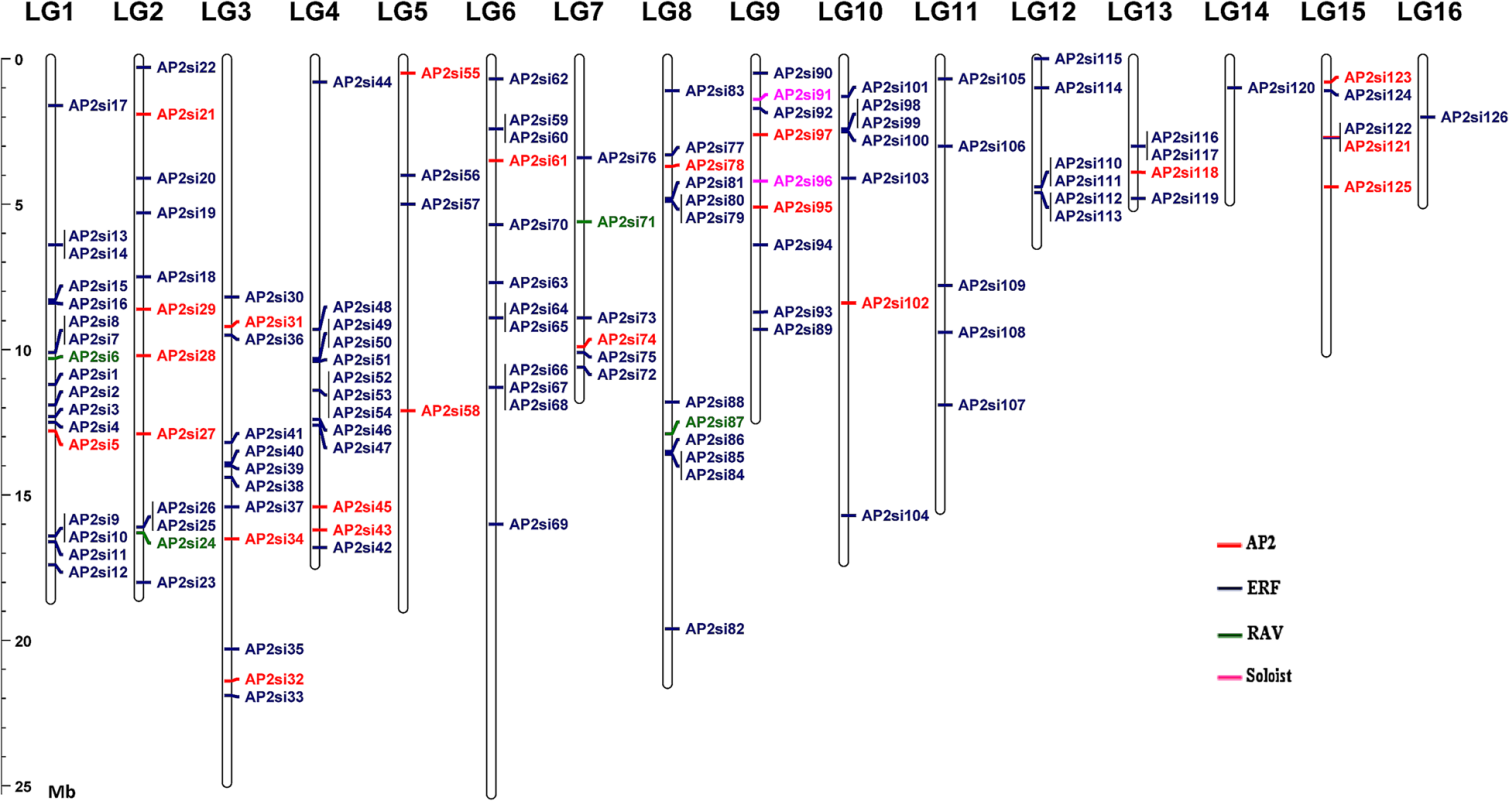
Mapping of sesame AP2/ERF genes based on their physical positions. Vertical bars represent linkage groups (LG) of the sesame genome. The LG numbers are indicated at the top of each LG. The scale on the left is in megabases

The localization of the AP2/ERF genes revealed that they are distributed unevenly distributed on the 16 Linkage Groups (LGs). The precise position (in bp) of each AP2/ERF on the sesame LGs is detailed in Additional file 1. Six genes (*AP2si127*, *AP2si128*, *AP2si129*, *AP2si130*, *AP2si131* and *AP2si132*) were not mapped because they were located on the unanchored scaffolds (Table 2). The largest number of genes (17; 12.88 %) was found on LG1, whereas LG14 and LG16 have only one gene (0.76 %). The two “Soloist” genes were mapped on the same LG9 (Fig. 2). The distribution pattern of these genes on some LGs pointed out some regions with relatively high accumulation of AP2/ERF genes in cluster. This can be observed in the LG1, LG3, LG4, LG8 and LG12. In overall, each LG had a mixture of the different families except LG11 and LG12 which only contained ERF genes.

**Table 2:** Summary of the AP2/ERF genes identified in the sesame genome

Gene Name	Locus ID	Linkage Group	Family	Group	ORF length (bp)	Gene Name	Locus ID	Linkage Group	Family	Group	ORF length (bp)
*AP2si1*	*SIN_1009471*	LG1	ERF	B2	1176	*AP2si67*	*SIN_1018537*	LG6	ERF	B3	717
*AP2si2*	*SIN_1009557*	LG1	ERF	A4	678	*AP2si68*	*SIN_1018539*	LG6	ERF	A4	678
*AP2si3*	*SIN_1009621*	LG1	ERF	B5	564	*AP2si69*	*SIN_1020950*	LG6	ERF	B5	624
*AP2si4*	*SIN_1010767*	LG1	ERF	B5	537	*AP2si70*	*SIN_1023331*	LG6	ERF	B5	714
*AP2si5*	*SIN_1010804*	LG1	AP2		996	*AP2si71*	*SIN_1006144*	LG7	RAV		1059
*AP2si6*	*SIN_1013634*	LG1	RAV		1065	*AP2si72*	*SIN_1008777*	LG7	ERF	A2	1080
*AP2si7*	*SIN_1013660*	LG1	ERF	B5	1074	*AP2si73*	*SIN_1009173*	LG7	ERF	B5	1119
*AP2si8*	*SIN_1013661*	LG1	ERF	B5	678	*AP2si74*	*SIN_1009317*	LG7	AP2		1971
*AP2si9*	*SIN_1013877*	LG1	ERF	B3	546	*AP2si75*	*SIN_1009337*	LG7	ERF	A2	600
*AP2si10*	*SIN_1013878*	LG1	ERF	B3	576	*AP2si76*	*SIN_1011553*	LG7	ERF	A2	639
*AP2si11*	*SIN_1013899*	LG1	ERF	A4	861	*AP2si77*	*SIN_1006545*	LG8	ERF	B6	1038
*AP2si12*	*SIN_1014016*	LG1	ERF	B6	819	*AP2si78*	*SIN_1006598*	LG8	AP2		621
*AP2si13*	*SIN_1017801*	LG1	ERF	A1	564	*AP2si79*	*SIN_1011988*	LG8	ERF	B3	474
*AP2si14*	*SIN_1017805*	LG1	ERF	A1	669	*AP2si80*	*SIN_1011989*	LG8	ERF	B3	501
*AP2si15*	*SIN_1017959*	LG1	ERF	B5	990	*AP2si81*	*SIN_1011991*	LG8	ERF	B3	441
*AP2si16*	*SIN_1017978*	LG1	ERF	A6	894	*AP2si82*	*SIN_1014868*	LG8	ERF	B4	837
*AP2si17*	*SIN_1021559*	LG1	ERF	A4	732	*AP2si83*	*SIN_1019697*	LG8	ERF	A6	1104
*AP2si18*	*SIN_1005434*	LG2	ERF	A5	450	*AP2si84*	*SIN_1026397*	LG8	ERF	A6	804
*AP2si19*	*SIN_1013217*	LG2	ERF	A5	729	*AP2si85*	*SIN_1026398*	LG8	ERF	B5	783
*AP2si20*	*SIN_1013368*	LG2	ERF	B4	1254	*AP2si86*	*SIN_1026408*	LG8	ERF	B4	768
*AP2si21*	*SIN_1017047*	LG2	AP2	A6	1146	*AP2si87*	*SIN_1026470*	LG8	RAV		1050
*AP2si22*	*SIN_1017255*	LG2	ERF	A4	525	*AP2si88*	*SIN_1026594*	LG8	ERF	B5	1257
*AP2si23*	*SIN_1018060*	LG2	ERF	B5	1173	*AP2si89*	*SIN_1005162*	LG9	ERF	A6	969
*AP2si24*	*SIN_1018312*	LG2	RAV		1125	*AP2si90*	*SIN_1010530*	LG9	ERF	A1	645
*AP2si25*	*SIN_1018336*	LG2	ERF	B5	1065	*AP2si91*	*SIN_1010676*	LG9	Soloist		684
*AP2si26*	*SIN_1018337*	LG2	ERF	B5	636	*AP2si92*	*SIN_1010728*	LG9	ERF	B6	837
*AP2si27*	*SIN_1021103*	LG2	AP2		1191	*AP2si93*	*SIN_1014969*	LG9	ERF	B4	1134
*AP2si28*	*SIN_1023829*	LG2	AP2		1473	*AP2si94*	*SIN_1024041*	LG9	ERF	A1	660
*AP2si29*	*SIN_1023944*	LG2	AP2		1062	*AP2si95*	*SIN_1024170*	LG9	AP2		1404
*AP2si30*	*SIN_1010313*	LG3	ERF	A4	648	*AP2si96*	*SIN_1024299*	LG9	Soloist		669
*AP2si31*	*SIN_1010442*	LG3	AP2		1680	*AP2si97*	*SIN_1024507*	LG9	AP2		1377
*AP2si32*	*SIN_1011351*	LG3	AP2		1674	*AP2si98*	*SIN_1001618*	LG10	ERF	B3	393
*AP2si33*	*SIN_1011410*	LG3	ERF	A5	468	*AP2si99*	*SIN_1001619*	LG10	ERF	B3	405
*AP2si34*	*SIN_1015264*	LG3	AP2		1779	*AP2si100*	*SIN_1001620*	LG10	ERF	B3	711
*AP2si35*	*SIN_1015746*	LG3	ERF	A6	993	*AP2si101*	*SIN_1010169*	LG10	ERF	B5	1029
*AP2si36*	*SIN_1017548*	LG3	ERF	B2	1182	*AP2si102*	*SIN_1017733*	LG10	AP2		1026
*AP2si37*	*SIN_1021800*	LG3	ERF	B3	642	*AP2si103*	*SIN_1019010*	LG10	ERF	A6	1218
*AP2si38*	*SIN_1021880*	LG3	ERF	B5	570	*AP2si104*	*SIN_1026203*	LG10	ERF	B2	810
*AP2si39*	*SIN_1021925*	LG3	ERF	A6	873	*AP2si105*	*SIN_1005694*	LG11	ERF	B5	948
*AP2si40*	*SIN_1021932*	LG3	ERF	B5	516	*AP2si106*	*SIN_1008520*	LG11	ERF	A4	759
*AP2si41*	*SIN_1022039*	LG3	ERF	B4	792	*AP2si107*	*SIN_1012983*	LG11	ERF	A1	567
*AP2si42*	*SIN_1001671*	LG4	ERF	B3	579	*AP2si108*	*SIN_1024656*	LG11	ERF	A5	675
*AP2si43*	*SIN_1001908*	LG4	AP2		2094	*AP2si109*	*SIN_1024820*	LG11	ERF	A4	732
*AP2si44*	*SIN_1006214*	LG4	ERF	A2	867	*AP2si110*	*SIN_1005299*	LG12	ERF	B1	451
*AP2si45*	*SIN_1007132*	LG4	AP2		1338	*AP2si111*	*SIN_1005300*	LG12	ERF	B1	753
*AP2si46*	*SIN_1012110*	LG4	ERF	A2	1095	*AP2si112*	*SIN_1005329*	LG12	ERF	B3	435
*AP2si47*	*SIN_1012139*	LG4	ERF	A3	1023	*AP2si113*	*SIN_1005330*	LG12	ERF	B3	795
*AP2si48*	*SIN_1016446*	LG4	ERF	B5	1197	*AP2si114*	*SIN_1014405*	LG12	ERF	B1	708
*AP2si49*	*SIN_1016588*	LG4	ERF	A1	507	*AP2si115*	*SIN_1014559*	LG12	ERF	A6	1059
*AP2si50*	*SIN_1016589*	LG4	ERF	A1	678	*AP2si116*	*SIN_1003959*	LG13	ERF	A5	369
*AP2si51*	*SIN_1016611*	LG4	ERF	B6	930	*AP2si117*	*SIN_1003961*	LG13	ERF		585
*AP2si52*	*SIN_1016730*	LG4	ERF	B3	690	*AP2si118*	*SIN_1004087*	LG13	AP2		1515
*AP2si53*	*SIN_1016731*	LG4	ERF	B3	963	*AP2si119*	*SIN_1004332*	LG13	ERF	A6	1110
*AP2si54*	*SIN_1016732*	LG4	ERF	B3	684	*AP2si120*	*SIN_1004964*	LG14	ERF	B1	684
*AP2si55*	*SIN_1005501*	LG5	AP2		1395	*AP2si121*	*SIN_1004866*	LG15	AP2		1545
*AP2si56*	*SIN_1007557*	LG5	ERF	A5	660	*AP2si122*	*SIN_1004869*	LG15	ERF	B4	273
*AP2si57*	*SIN_1013461*	LG5	ERF	A4	807	*AP2si123*	*SIN_1007981*	LG15	AP2		1152
*AP2si58*	*SIN_1023649*	LG5	AP2		789	*AP2si124*	*SIN_1008036*	LG15	ERF	B6	1140
*AP2si59*	*SIN_1009676*	LG6	ERF	B3	492	*AP2si125*	*SIN_1025656*	LG15	AP2		1950
*AP2si60*	*SIN_1009677*	LG6	ERF	B3	720	*AP2si126*	*SIN_1016948*	LG16	ERF	B1	666
*AP2si61*	*SIN_1009815*	LG6	AP2		1026	*AP2si127*	*SIN_1002608*	NA	ERF	B3	525
*AP2si62*	*SIN_1012570*	LG6	ERF	B2	756	*AP2si128*	*SIN_1002609*	NA	ERF	B3	801
*AP2si63*	*SIN_1015461*	LG6	ERF	B5	1071	*AP2si129*	*SIN_1002610*	NA	ERF	B3	648
*AP2si64*	*SIN_1015594*	LG6	ERF	A4	532	*AP2si130*	*SIN_1002670*	NA	ERF	A1	612
*AP2si65*	*SIN_1015595*	LG6	ERF	A1	630	*AP2si131*	*SIN_1000334*	NA	AP2		1182
*AP2si66*	*SIN_1018534*	LG6	ERF	B3	765	*AP2si132*	*SIN_1000313*	NA	AP2		1182

### Phylogenetic analysis and mapping of orthologous genes

**Fig. 3:**
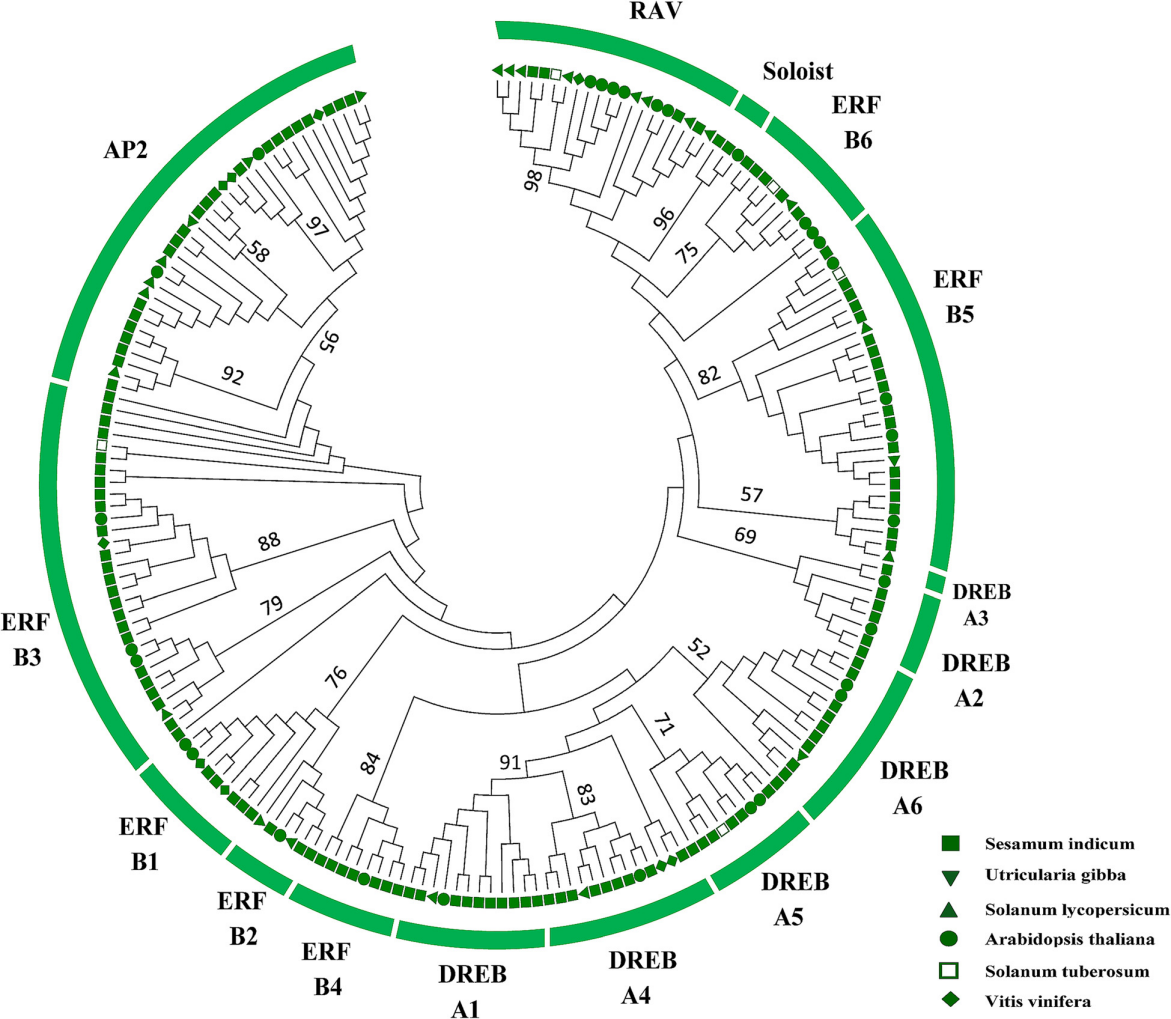
Maximum likelihood tree of AP2/ERF proteins in sesame, *Arabidopsis*, *Utricularia gibba*, grape, potato and tomato. Bootstrap values ≥ 50 % are shown

Two Maximum Likelihood (ML) trees were constructed, the first resulting from the alignments of only AP2/ERF domains of the 132 protein sequences in sesame; the second ML tree resulted from the alignments of 202 full length protein sequences including 132 AP2/ERF in sesame and 70 protein sequences selected from each family of AP2/ERF reported in the 5 relative species (12 in tomato, 13 in *U. gibba*, 5 in potato, 31 in *Arabidopsis* and nine in grape). In the first ML tree, all genes of AP2 family were clearly distinguished from those of the ERF family. The RAVs and Soloists appeared to be more close to the AP2 family (Additional file 2). The second ML tree was constructed to precisely dissect the functional groups within each subfamily according to *Arabidopsis* AP2/ERF genes which have been investigated extensively. The un-rooted tree divided the AP2/ERF genes into 15 major groups (Fig. 3). We found 6 groups (A1-A6 and B1-B6) within DREB and ERF subfamilies, respectively. In contrast to the DREB subfamily groups which clustered together, strangely, the ERF subfamily genes formed two clades intervened by DREB: one gathered B1, B2, B3, and B4 and the other gathered B5 and B6. The number of genes belonging to each group is reported in the Table 1 and more in details in Table 2.

**Fig. 4:**
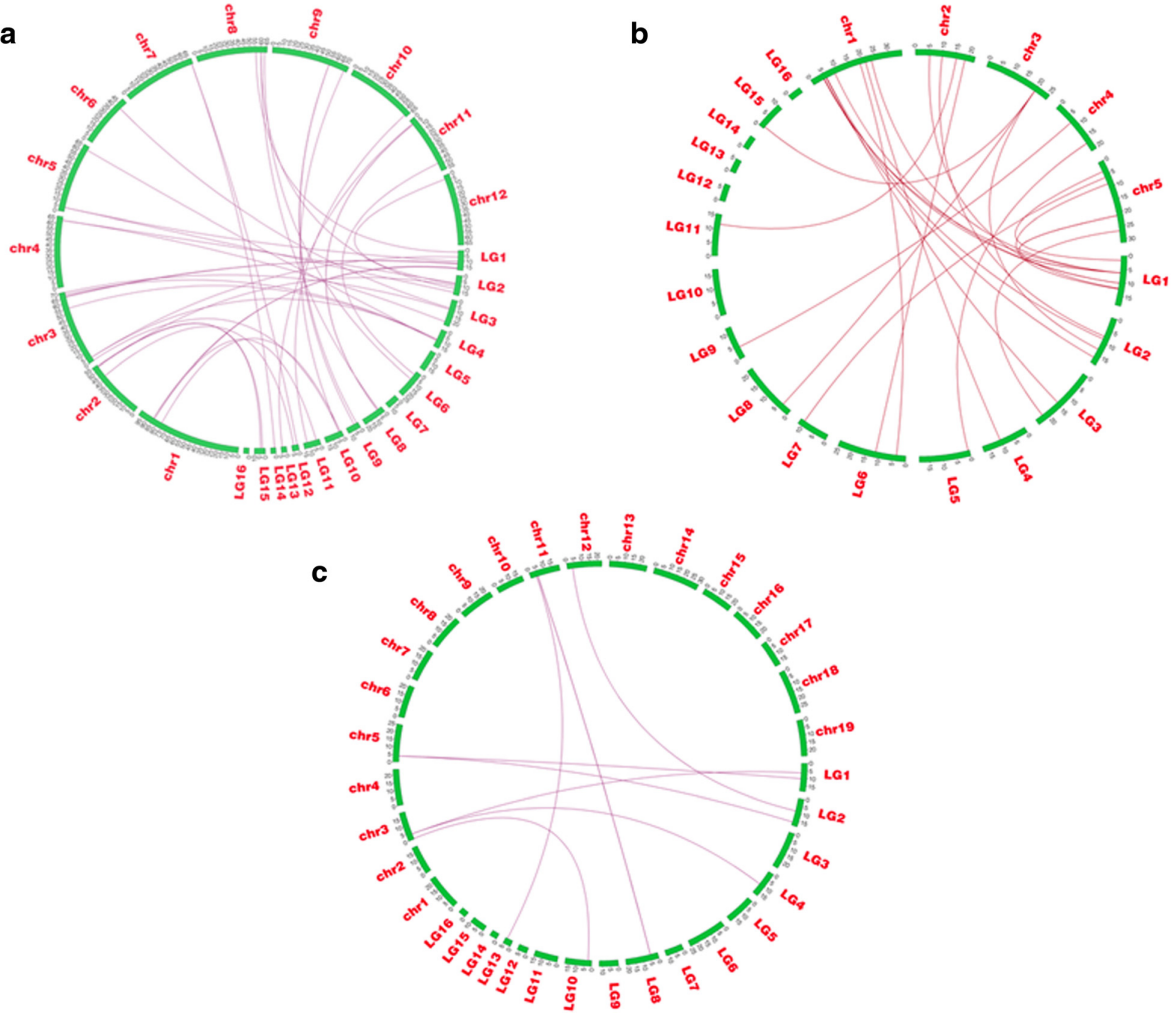
Orthologous relationships of AP2/ERF genes between **a** sesame and *Arabidopsis*; **b** sesame and tomato; **c** sesame and grape genomes. Green bars represent the chromosomes of each pair of species. “LG” represents the linkage groups of sesame genome and “Chr” represents the chromosomes of the other species genomes

In addition, we performed a genome-wide comparative analysis to identify the orthologous AP2/ERF transcription factors between sesame, *Arabidopsis*, grape and tomato (Fig. 4). Largest orthology of AP2/ERF genes in sesame was found with tomato (38) followed by *Arabidopsis* (24) and least with grape (13). The orthologous gene pairs and localization in each genome are presented in Additional file 3. All the four families were represented in the orthologous gene pairs and distributed throughout all the LGs except the LG16. Out of the 24 gene pairs between sesame and *Arabidopsis*, 15 of *Arabidopsis* AP2/ERF genes retained one copy, three genes (*AT1G13260*, *AT1G15360* and *AT5G51990*) retained two copies and only one gene (*AT3G54320*) conserved a tripled copy in sesame genome. Inversely, two genes in sesame (*AP2si6* and *AP2si13*) preserved two copies and 22 genes retained one copy in *Arabidopsis* genome. In summary, 22 AP2/ERF genes in sesame have 15 corresponding genes in *Arabidopsis* genome. When compared with tomato, it was revealed that nine genes retained two copies while 20 genes retained one copy in the sesame genome. Similar to *Arabidopsis*, orthologous genes of grape also showed the retention of one, two and three copies patterns of genes in sesame genome. Interestingly, four sesame genes (*AP2si27*, *AP2si29*, *AP2si61* and *AP2si78*) belonging to the AP2 family, found their orthologous counterpart in tomato, grape and *Arabidopsis* at once. In overall, the results of the orthologs analysis and the phylogenetic relationships between sesame and its relatives were consistent with some orthologous genes found to be closely located in the tree.

Based on the accumulated evidences indicating that the AP2/ERF proteins are involved in various abiotic stress responses and then could help in marker-aided breeding, we performed SSR search in all of AP2/ERF genes in sesame. The analysis yielded 91 SSR markers distributed throughout the LGs. Twelve genes yielded two SSRs and no SSR marker was found in 47 AP2/ERF genes (Additional file 4). Surprisingly, only two SSR motif types were retrieved including trinucleotide motif (90.91 %) and hexanucleotide motif (8.91 %). These markers developed, would be useful in genotyping and MAS for sesame improvement towards abiotic stresses.

### Gene structure and conservative motifs distribution analysis of AP2/ERF genes

**Fig. 5:**
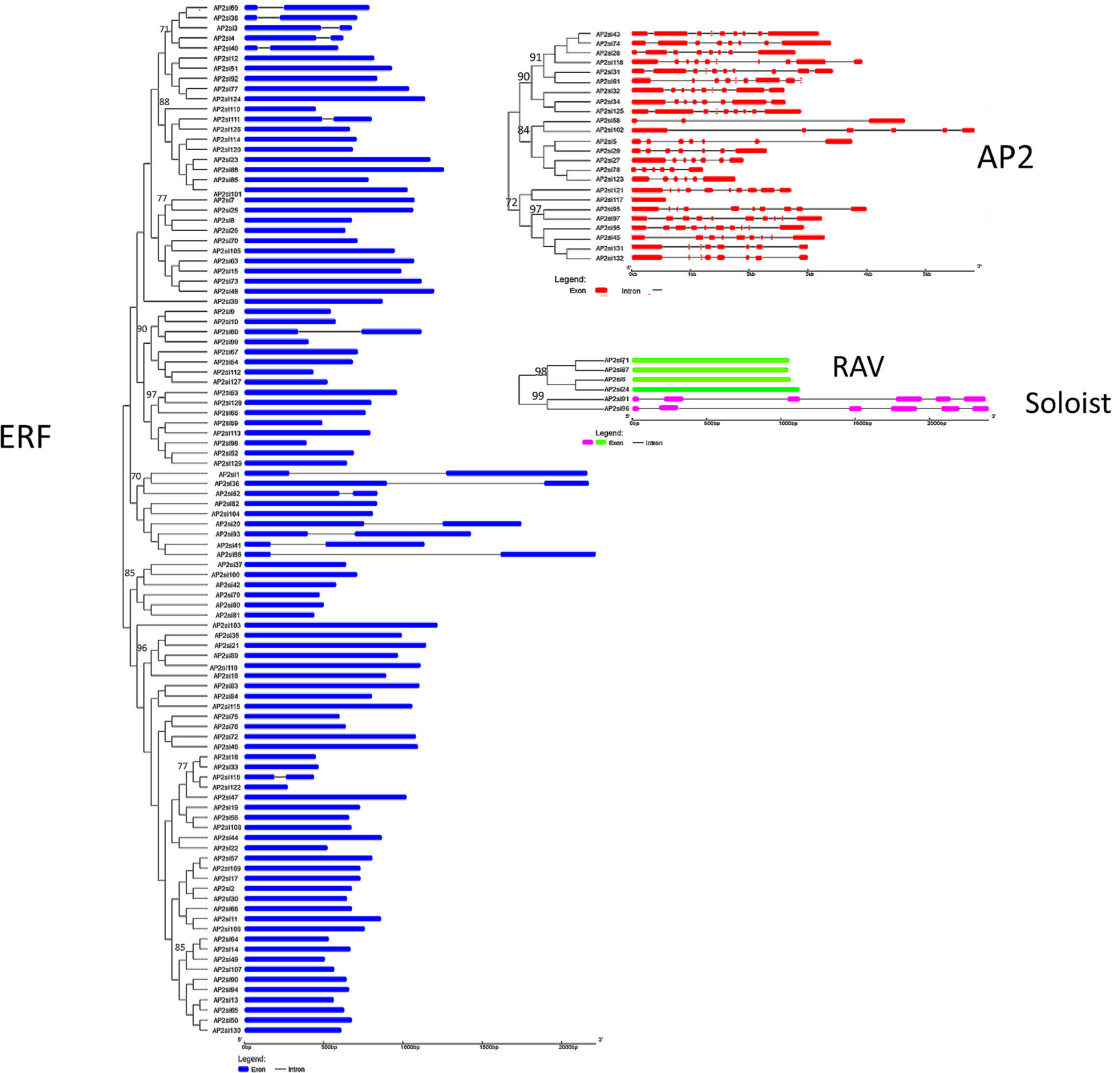
Gene structures of 132 AP2/ERF proteins according to each family. Exons and introns are represented by colored boxes and black lines, respectively

To gain insights into the structural diversity of the AP2/ERF genes, we constructed a phylogenetic tree with the full length protein sequences of the four families and displayed the exon/intron organization in the coding sequences by comparing their ORFs with their genomic sequences (Fig. 5). Sesame AP2/ERF genes contained 1 to 10 exons with nearly 70 % of intronless genes. The schematic structures revealed that most of the ERF genes have 1 exon except the genes *AP2si1*, *AP2si3*, *AP2si4*, *AP2si20*, *AP2si36*, *AP2si38*, *AP2si40*, *AP2si41*, *AP2si60*, *AP2si62*, *AP2si69*, *AP2si86*, *AP2si93*, *AP2si111* and *AP2si116* which have exactly two exons. The four RAV genes also possess only one exon with similar lengths. Unlike the ERF genes, the coding sequences of the AP2 genes are disrupted by many introns with the number of exons ranging from three (*AP2si58*) to ten (*AP2si121*, *AP2si118*, *AP2si97*, *AP2si95*, *AP2si55*, *AP2si31*). One exceptional case is the gene *AP2si117* which displayed only one exon. Finally, the two “Soloist” genes showed exactly six exons, distributed in similar regions of the genes. Besides the consistency with the phylogenetic analysis, we found that the genes that clustered in the same group displayed similar exon–intron structures, differing only in intron and exon lengths. This can be observed in the first clade of ERF which gathered 5 genes (*AP2si3*, *AP2si4*, *AP2si38*, *AP2si40* and *AP2si69*) displaying 2 exons. However, this is not the case for all close gene pairs. For instance, the gene *AP2si117* with only one exon occurred in the same cluster with the genes *AP2si45*, *AP2si55*, *AP2si95*, *AP2si97*, *AP2si131*, *AP2si132* and *AP2si121* which displayed more than eight exons.

**Fig. 6:**
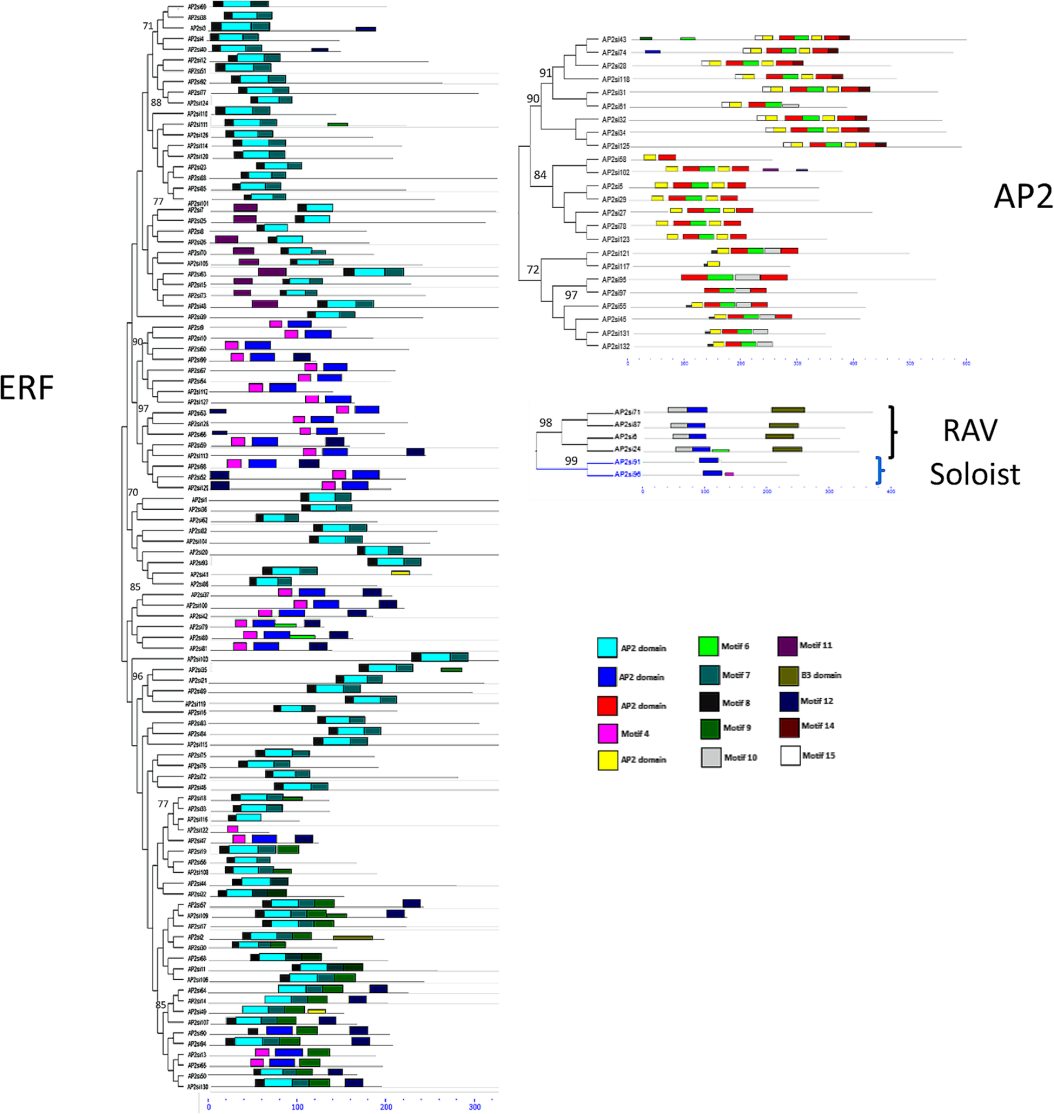
Motifs identified by MEME tools in sesame AP2/ERF protein sequences according to each family. Fifteen motifs were identified and indicated by different colors. Motif location was showed

In addition, to investigate the motifs shared by related proteins in the different families, the MEME motif search tool was employed and the motifs found were then subjected to SMART annotation and confirmed in Pfam database. In total, 15 conserved motifs were identified, lengths ranging from 11-50 amino acids (Additional file 5). The motifs 1, 2, 3 and 5 specifying the AP2 domain were identified in all the 132 AP2/ERF proteins while the motif 12 related to B3 domain was found in the four RAV genes (Additional file 6). However, the remaining motifs were unidentified when searched by SMART and Pfam databases. We further analyzed the motifs other than the AP2/ERF conserved domain existing in some ERF/DREB functional groups based on the conserved motifs described by Nakano et al. [26]. The results showed that although small amino-acids vary slightly, sesame ERF/DREB groups are characterized by the same conserved motifs identified by Nakano et al. [26] in Arabidopsis and rice (Additional file 7). This indicated the good conservation of this gene family in plant species. The phylogenetic tree and the motifs dissection results were consistent because most of the closely related members in the phylogenetic tree had common motifs composition and organization (Fig. 6).

### Tissue-specific expression profiling of AP2/ERF genes and drought stress responses of DREB subfamily genes

**Fig. 7:**
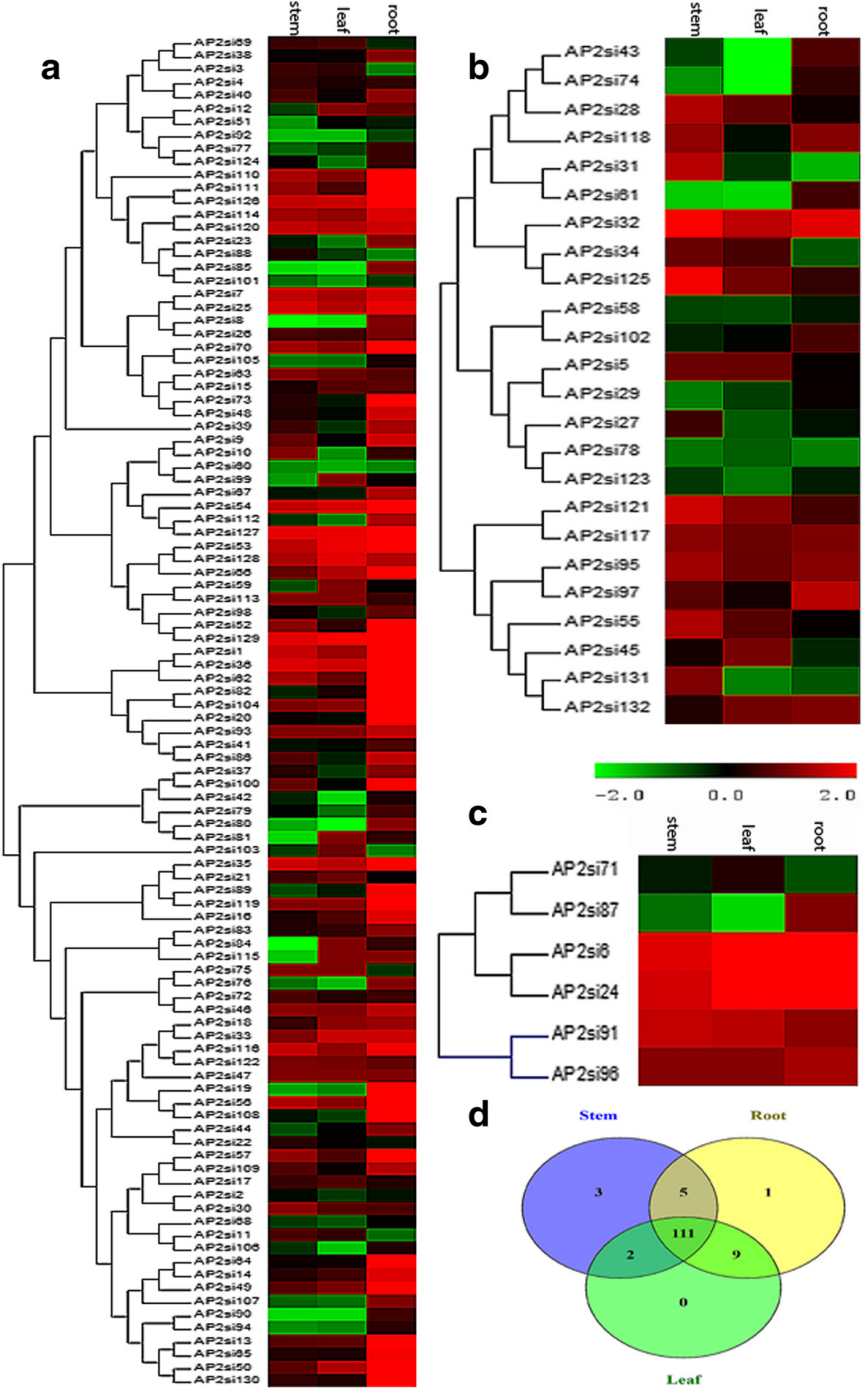
Expression profile analysis of AP2/ERF genes in sesame tissues according to each family. **a** ERF. **b** AP2 family **c** RAV and Soloist families **d** Venn diagram depicting the distribution of shared expressed AP2/ERF genes among sesame tissues. Transcriptome data were used to measure the expression level of AP2/ERF genes in roots, stem tip and leaves. The color scale for expression values is shown

Transcriptome data from three tissue samples namely root, leaf and stem were used for identifying genes differentially expressed in these tissues. Heat maps were generated according to the different AP2/ERF subfamilies based on the RPKM values for each gene in all tissue samples (Fig. 7). Apart from AP2si47 gene that was not expressed across the tissues, all AP2/ERF genes displayed very diverse expression. In general, it is observed that gene expression patterns were almost conserved within subfamilies, although expression levels of specific members could be changed from tissue to tissue. The ERF family exhibited the highest expression in all tissues. Similarly, high expressions of two members (*AP2si6* and *AP2si24*) of the RAV family were shown in all tissues while the two remaining members displayed a relatively low expression level. The AP2 family expression levels were lower than most of other AP2/ERF genes. In general, majority of genes displayed a higher expression in the root compared to other tissues. Furthermore, 84.73 % of TFs (111) were expressed in all tissues suggesting a control of a broad set of genes at transcriptional level. The AP2 genes *AP2si131*, *AP2si31* and *AP2si27* exhibited stem-specific expression; the ERF gene *AP2si115* exhibited root-specific expression while no specific gene was expressed in leaf (Fig. 7). The genes *AP2si6* and *AP2si24* (RAV family); *AP2si36*, *AP2si54*, *AP2si127* and *AP2si129* (ERF subfamily) were found to be constitutively expressed at a relatively high levels in all the three tissues.

**Fig. 8:**
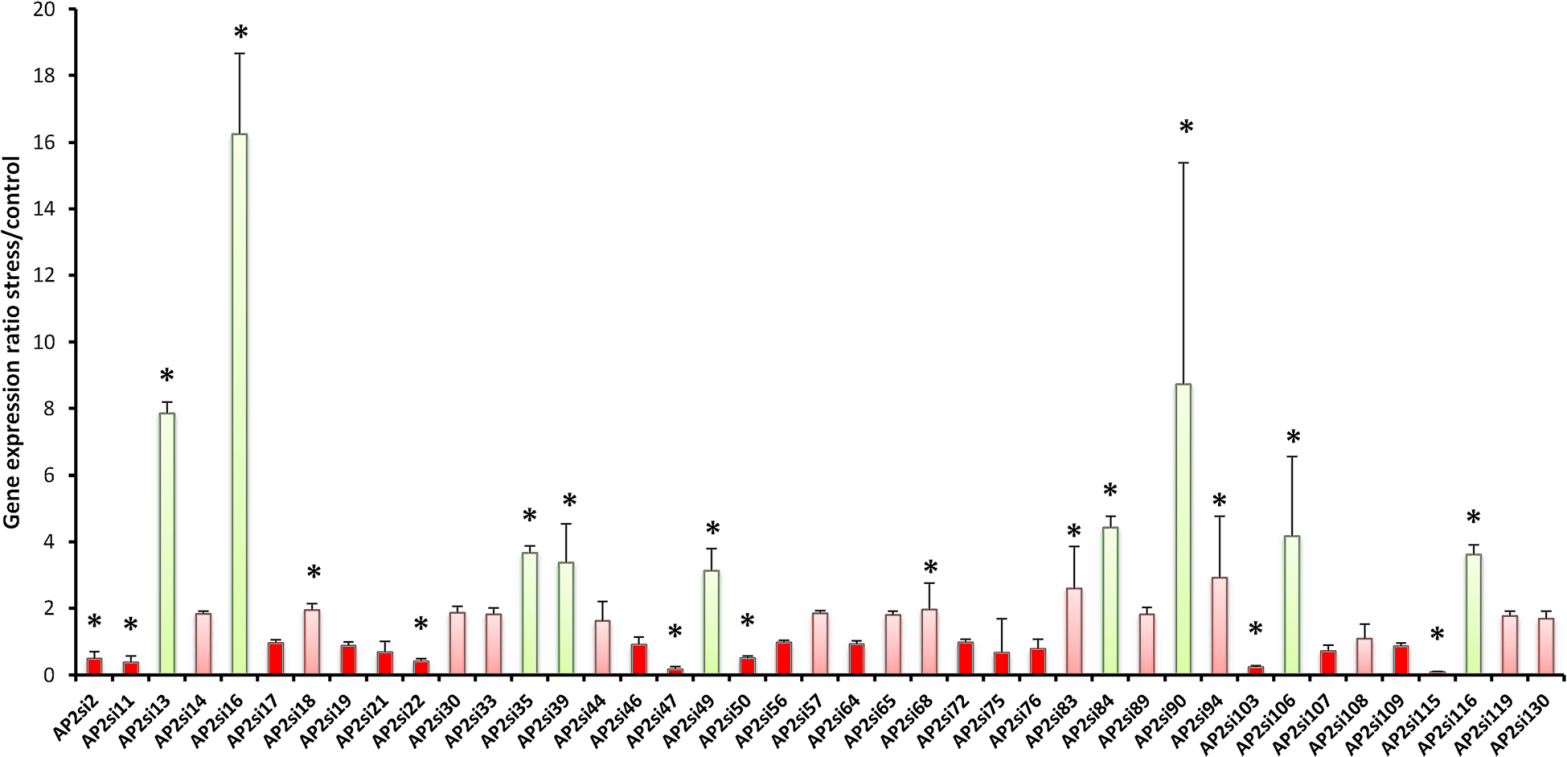
DREB genes induction rates in sesame roots during 3 days of drought stress in comparison to control condition. Transcripts abundance was quantified through qRT-PCR and the experimental values were normalized using sesame *actin7* as reference gene. The mean values issued from three independent biological replicates were analysed for significance using the statistical *t*-test (*p* value <0.01). The histograms represent the relative expression values of induction rates (stress/control). The green bars represent the most up-regulated genes, the pink bars represent the genes moderately up-regulated and the red bars represent the down-regulated genes. * means significant 2-fold rate increase in gene expression (stress/control)

qRT-PCR was used to analyze the expression profiles of DREB genes under drought stress condition. As shown in Fig. 8, an overall differential expression patterns were observed among the genes. Twenty-three DREB genes were up-regulated under drought stress including 13 genes with more than 2-fold rate increase of expression level (*p* value <0.01), suggesting that these genes might play some important roles in the regulation of drought stress in sesame. More remarkably, the gene *AP2si16* belonging to the DREB6 group, significantly exhibited the highest expression level with more than 16-fold rate increase. The genes *AP2si90* (DREB1), *AP2si13* (DREB1), *AP2si84* (DREB6), *AP2Si106* (DREB4), *AP2si35* (DREB6), *AP2si116* (DREB5), *AP2si49* (DREB1) and *AP2si39* (DREB6) also displayed strong expression levels (from 3 to 8-fold rate increase). In contrast, the 3-days of drought stress has decreased the transcript abundance of 18 (44 %) DREB genes. Four genes namely *AP2si115* (DREB6), *AP2si47* (DREB3), *AP2si103* (DREB6) and *AP2si11* (DREB4) were the most repressed ones with more than 10-fold decrease of expression levels. In overall, DREB groups show acute responses to drought and might related to sesame drought tolerance knowing that the material used in the qRT-PCR is a strong drought tolerant accession.

## Discussion

In this study, 132 AP2/ERF family genes were identified in the sesame genome. Compared to the five related species, sesame harbors the lowest number of AP2/ERF genes. Sesame was estimated to have diverged from the tomato-potato lineage approximately 125 MYA (million years ago) and from *U. gibba* approximately 98 MYA [40]. Moreover, genome analysis showed that both *U. gibba* and sesame had undergone recent duplication events (WGD). The relatively low number of AP2/ERF genes found in sesame genome is surprising, knowing on one hand that, sesame is relatively tolerant to drought and many other abiotic stresses [44] compared to the 5 related species and in the other hand, the role of AP2/ERF genes in response to abiotic stresses in plants. This suggests the possibility of a gene loss event, which often follows WGD, during the evolutionary process of sesame. Similar assumptions were posited in castor bean which also naturally displays a strong tolerance to diverse environmental stresses but contains small AP2/ERF superfamily members [31]. We further compared the members of each subfamily between sesame and *U. gibba* and found that genes loss might occur mainly in ERF and DREB subfamilies.

According to the classification of [19], the AP2 family members should have had two AP2/ERF domains. However, in this study, it was discordant that 4 genes namely *AP2si132*, *AP2si117*, *AP2si58* and *AP2si131* with only one AP2/ERF domain were classified in the same group as the “real” AP2 family members. Recently, many authors reported similar results regarding the AP2 family members with only one AP2/ERF domain (four genes found in *Arabidopsis* [26]; five in tomato [42]; seven in hevea [30]; five in *Brassica rapa* [45]; three in switchgrass [46]. This implies more detailed analysis in the AP2 family is needed for a new classification approach.

Using phylogeny approach may afford insights into genes function and facilitate the identification of orthologous genes assuming that, genes with conserved functions show a tendency to cluster together. This approach has been widely applied for prediction of the functions of AP2/ERF proteins in many plant species such as grape, foxtail millet, *Brassica*, rice [33]. The proximity of the RAV and Soloist genes to the AP2 family found in this study was recently reported in switchgrass [46]. Moreover, the ML tree based on the AP2/ERF protein sequences of the 6 species displays particular pattern with 2 clades of the ERF subfamily groups failing to cluster together as found by Song et al. [32]; Lata et al. [33]; Rao et al. [22]. Further in-depth *in silico* analysis is requisite for finding the possible reasons for such observations [33].

Based on the phylogenetic tree, different functions could be assigned to the AP2/ERF groups in sesame. For instance, the group B6 including five sesame ERF genes, clusters together with the *Arabidopsis* gene RAP2.11 known to be involved in plant response to low-potassium conditions [47]. We speculated that these five genes might have similar functions. The Group A2 included five sesame genes (*AP2si44*, *AP2si46*, *AP2si72*, *AP2si75* and *AP2si76*) and was close to the well-studied gene DREB2A in *Arabidopsis* involved in responses to water stress and heat stress [34]. Hence, it is possible to hypothesize that these genes might be involved in similar activities. Likewise, individual gene function could also be predicted based on the close relationships between sesame and *Arabidopsis* through the homolog-based gene function prediction. For example, the gene *AP2si55* belonging to the AP2 family might play similar role as its ortholog *AT4G36920* from *Arabidopsis* involved in the floral identity specification as well as development of the ovule and seed coat [48, 49].

Gene expression patterns can also provide important clues for gene function prediction [50]. The tissue-specific expression profiling showed that most of the AP2/ERF genes are expressed in all sesame tissues analyzed. However, a higher expression was detected in sesame root and similar results were reported in castor bean [31], Chinese cabbage [32] and foxtail millet [33]. The ERF, RAV and Soloist family members displayed higher expression in sesame tissues than AP2 family members indicating that these families might play a central role in tissues development and sesame plant growth [32].

The variability in expression patterns of sesame DREB genes observed in this study indicated that they might be involved in different regulation pathways for drought stress response. Moreover, we observed that the genes from the same group could be expressed differently in response to drought stress and, therefore, are thought to have different functions. Expression analyses of DREB genes also showed unusual and plausible roles for some group members during drought stress. This is the case of the DREB6 group members which functions are scarcely reported in the literature [51]. Out of the ten members of this group, seven were highly up-regulated, pinpointing their importance in drought stress response in sesame. In the same line, the DREB1 genes are mostly known as cold response genes [52, 53]; however, as reported by some authors [53–56] and confirmed in our study (seven genes up-regulated *vs* three down-expressed), these genes are highly involved in drought stress response pathways in sesame knowing that sesame is not cold areas crop but grown in arid and semi-arid areas. Hence, these functional DREB groups might probably participate in the relative drought tolerance naturally exhibited by sesame. Intriguingly, it is noteworthy that, the DREB2 genes which are well described in many crops as actively involved in drought response pathways [35, 36, 57, 58] do not seem to be highly expressed in our study. This uncommon feature may indicate that this group’s members might be involved principally in the regulation of other stress transduction pathways in sesame. Knowing sesame as a survivor crop mainly grown in marginal areas with the occurrence of high temperatures and frequent drought, we may hypothesize from our results that, sesame has probably oriented and dedicated a large part of its DREB group’s members to regulate its main abiotic stresses especially drought. The strongly up-regulated gene identified in this study (*AP2si16*) is the ortholog of *AT1G64380* in *Arabidopsis*, described as responsive to the chitin treatment, a main elicitor of the plant defense response against pathogens [59]. This indicates possible new functions of this gene which plays essential role in abiotic stress tolerance in plant and may be an excellent candidate for the engineering of sesame breeding with improved drought stress tolerance.

## Conclusions

To the best of our knowledge, no study has been conducted on the AP2/ERF superfamily in sesame to date. Therefore, this is the first comprehensive study on these TFs in sesame aiming to help elucidating the genetic basis for the stress adaptation of sesame especially for drought tolerance. One hundred and thirty two AP2/ERF genes were identified in the sesame genome including all families previously reported in the AP2/ERF superfamily. In addition, the expression patterns described together with the comparison of homologs from other species can provide a basis for identifying the roles of the different members of sesame AP2/ERF genes. Hence, further works should rely on these gene resources to characterize candidate genes to improve tolerance to major abiotic constraints of sesame production.

## Methods

### Data resources and AP2/ERF superfamily transcription factor identification in sesame

AP2/ERF genes and proteins sequences of *Arabidopsis thaliana, Vitis vinifera, Solanum lycopersicum, Solanum tuberosum* and *Utricularia gibba* were downloaded from the Plant Transcription Factor DataBase (http://planttfdb.cbi.pku.edu.cn/) [41]. In addition, the sesame genome and proteome were downloaded from the Sinbase (http://ocri-genomics.org/Sinbase/) [60]. The phylogeny data of the six species were downloaded from NCBI Taxonomy common tree (http://www.ncbi.nlm.nih.gov/Taxonomy/CommonTree/wwwcmt.cgi).

The Hidden Markov Model (HMM) profile of the AP2/ERF domain (PF00847) was obtained from Pfam v28.0 database (http://pfam.xfam.org/) [61] and searched against the sesame proteome using Unipro UGENE [62]. A total of 132 AP2/ERF proteins were obtained as candidate AP2/ERF genes. To further confirm these candidate genes, their amino acid sequences were explored on the Pfam database (http://pfam.xfam.org/search) and the Simple Modular Architecture Research Tool (SMART) [63] based on the conserved domain, to ensure the presence of AP2/ERF domain in each candidate protein.

### Chromosomal location, Gene structure and Motif identification of AP2/ERF genes

The physical positions of the identified AP2/ERF genes in sesame were searched on the Sinbase and mapped onto the 16 Linkage Groups (LGs) of sesame genome using MapChart 2.3 [64]. A structural figure of sesame AP2/ERF genes, including the numbers and locations of the exon and intron, was constructed based on Sinbase information and displayed using the Gene Structure Display Server (GSDS 2.0) web-based bioinformatics tool (http://gsds.cbi.pku.edu.cn/) [65]. The motif identification of sesame AP2/ERF protein sequences was performed using a motif-based sequence analysis tool, MEME Suite version 4.10.2 [66] with the following parameters: the optimum width of amino acid sequences was set from 6 to 50, the maximum number of motifs to 15, the number of repetitions to “any number” and all other parameters set at default. The amino acid sequences of the 15 motifs identified by MEME Suite were searched on Pfam database to find out the AP2/ERF motifs and their sequences logo were generated.

### Alignment, phylogenetic analysis and identification of microsatellite markers in sesame AP2/ERF genes

A single alignment of sesame AP2/ERF domain sequences and a multiple alignment analysis of the amino acid sequences of the AP2/ERF genes in sesame, *Arabidopsis*, grape, tomato, potato and *Utricularia gibba* were conducted using the Clustal W program built in the MEGA 6.0 software [67] with a gap open penalty of 10 and gap extension penalty of 0.2. Alignments were displayed using BoxShade (http://www.ch.embnet.org/software/BOX_form.html) (Additional file 8) and un-rooted Maximum-Likelihood (ML) trees were constructed in MEGA 6.0 software with a 1000 bootstrap value. Combining the phylogenetic trees with the conserved domain analysis, the AP2/ERF genes in sesame were classified into several subfamilies and groups according to [18]. Furthermore, the web based software Websat (http://wsmartins.net/websat/) [68] was used to identify simple sequence repeats (SSRs) in the predicted 132 AP2/ERF genes in sesame with the following parameters: two to six nucleotide motifs were considered, and the minimum repeat unit was defined as five reiterations for dinucleotides and four reiterations for other repeat units.

### Comparative mapping of orthologous AP2/ERF genes in sesame, *Arabidopsis*, tomato and grape

The amino acid sequences of the predicted AP2/ERF proteins were BLASTp searched against protein sequences of *Arabidopsis*, tomato and grape in NCBI. Hits with E-value ≥ 1e-40 and at least 75 % homology were considered significant [69]. The comparative orthologous relationships of AP2/ERF genes among the four species were illustrated using Circos program [70].

### Tissue-specific expression profiling using RNA-seq and qRT-PCR analysis of AP2/ERF genes under drought stress

To analyze the expression patterns of AP2/ERF genes in sesame, different transcriptome data from root, stem tip, and leaf previously obtained by our group were used. These data were downloaded from SesameFG (http://www.ncgr.ac.cn/SesameFG). The analysis were performed using Cluster3.0 (http://bonsai.hgc.jp/~mdehoon/software/cluster/software.htm), and reads per kilobase per million mapped reads (RPKM) values for each gene in all the tissue samples were log10 transformed. Finally, a heat map was generated by Multi Experiment Viewer (MEV) [71].

### Plant materials and stress treatment

A sesame accession highly tolerant to drought “ZZM5396” was obtained from the China National Genebank, Oil Crops Research Institute, Chinese Academy of Agricultural Sciences. Plants were grown in pots containing loam soil mixed with 10 % of added compound fertilizer and were kept in a greenhouse. The experiment was carried out in triplicate at the experimental field of Oil Crops Research Institute, Wuhan (China), with 3 plants kept per pot. The plants were regularly irrigated until early flowering stage and the drought stress was applied by withholding water for 3 days when the plant leaves began wilting. Meanwhile, the control plants were maintained under regular irrigation during all the experiment. The roots of the seedlings were harvested at the third day of stress for both stressed plants and control plants. The samples harvested from three individual plants and pooled were frozen immediately in liquid nitrogen and conserved in -80 °C until further use.

### RNA extraction and qRT-PCR analysis

Total RNA was isolated from roots of the 3-day stressed and unstressed sesame seedlings and cDNA library was constructed according to the procedure described by [72].

The DREB subfamily genes are widely reported to be involved in drought stress tolerance in many plants [56, 73]. Hence, this subfamily was retained for gene expression analysis under drought stress in our study. The specific primers for the 41 DREB genes were designed using the Primer Premier 5.0 [74] (Additional file 9). Expression of all sesame DREB subfamily genes was detected by qRT-PCR in triplicate and the sesame actin7 (*SIN_1006268*) gene was used as an internal control [1]. The 2^-ΔΔCt^ method was applied to calculate the change in expression of each gene [75].

### Statistical analysis

To analyze the statistical difference between the expressions of target genes, univariate analysis of variance (ANOVA) with *t*-test procedure was conducted using R 2.15.2, an open-source software.

## Abbreviations

AP2/ERF, APETALA 2/ethylene-responsive element binding factor; DREB, dehydration-responsive element binding protein; HMM, Hidden Markov Model; MAS, marker assisted selection, MEME, multiple em for motif elicitation; ML, maximum likelihood; MYA, million years ago; NCBI, National Center for Biotechnology Information; qRT-PCR, quantitative real time PCR; RAP2, related to AP2; RAV, related to ABI3/VP1; RPKM, reads per kilobase per million mapped reads; TF, transcription factor; WGD, whole-genome duplication; WGT, whole-genome triplication

## Additional files

Additional file 1: Characteristics of sesame AP2/ERF Transcription factor gene family members. This table contains the type, distribution of AP2 domains, protein full length, subfamily, ORF length, number of CDS, lists of predicted domains. (XLSX 43 kb) Additional file 2: ML tree of the 132 sesame AP2/ERF proteins. Bootstrap values ≥ 70 % are shown. (TIF 6890 kb) Additional file 3: Orthologous gene pairs of AP2/ERF and their localization in sesame, *Arabidopsis*, grape and tomato genomes. (DOCX 23 kb) Additional file 4: Details of sesame AP2/ERF transcription factor- based SSR markers. (XLSX 17 kb) Additional file 5: Motif sequences identified using MEME tools in sesame AP2/ERF genes. (DOCX 12 kb) Additional file 6: Sequence Logo of the 5 motifs corresponding to AP2/ERF domains. (TIF 7418 kb) Additional file 7: Identification of conserved motifs in some sesame ERF functional groups as described by Nakano et al. (2006) in Rice and Arabidopsis. A: DREB1. B: DREB3. C: DREB5. D: ERF1. E: ERF4. F: ERF5. (TIF 15878 kb) Additional file 8: Multiple alignments of AP2/ERF protein sequences. (PDF 182 kb) Additional file 9: List of primers used in quantitative real time- PCR analysis. (XLSX 12 kb)
