# Supplementary figures and images for: Insight into the AP2/ERF transcription factor superfamily in sesame and expression profiling of DREB subfamily under drought stress
Source: BMC Plant Biol. 2016 Jul 30;16:171. doi: 10.1186/s12870-016-0859-4 (PMC4967514; doi:10.1186/s12870-016-0859-4)

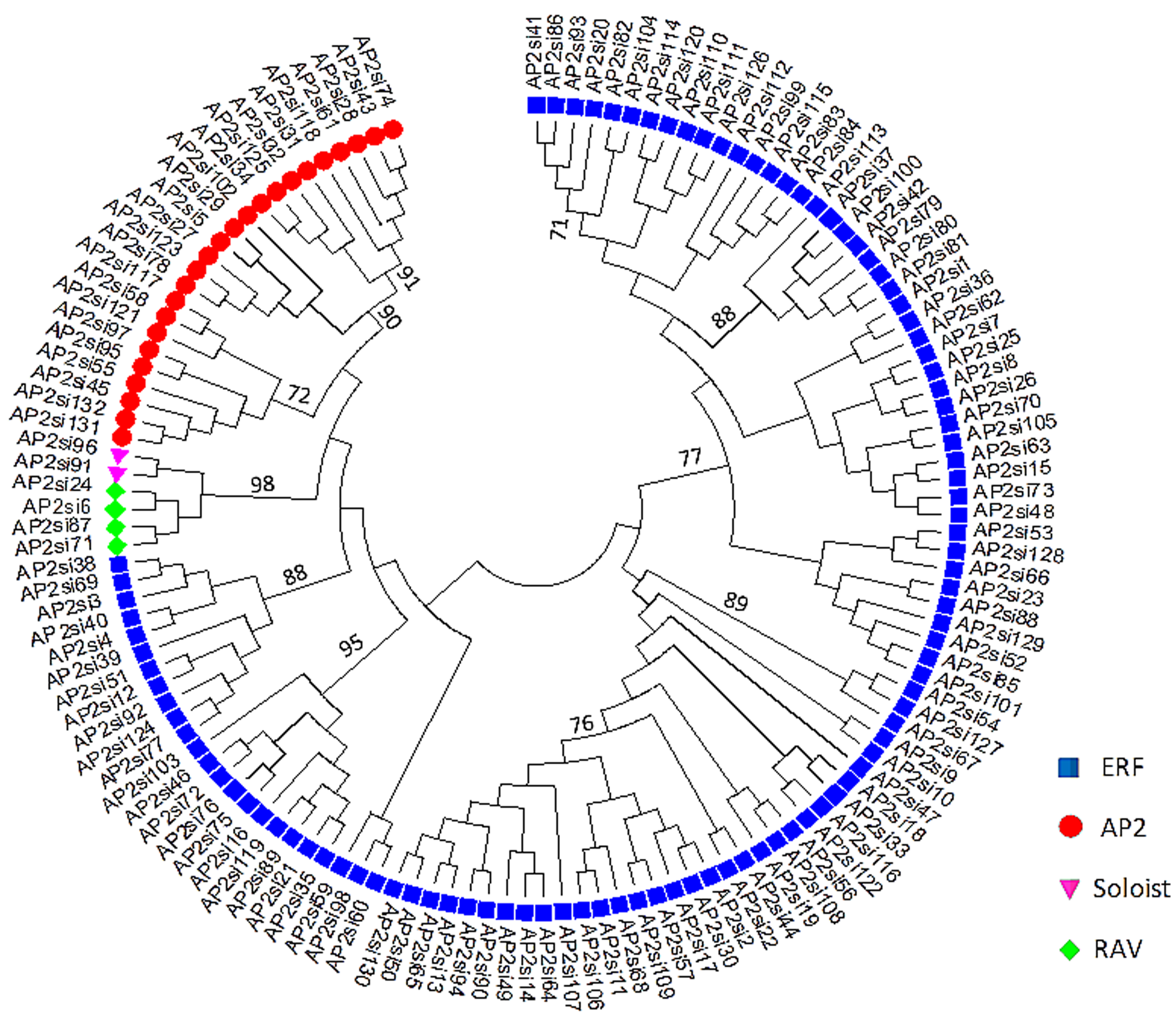

Supplement: Additional file 2: — ML tree of the 132 sesame AP2/ERF proteins. Bootstrap values ≥ 70 % are shown. (TIF 6890 kb) [file 12870_2016_859_MOESM2_ESM.tif]

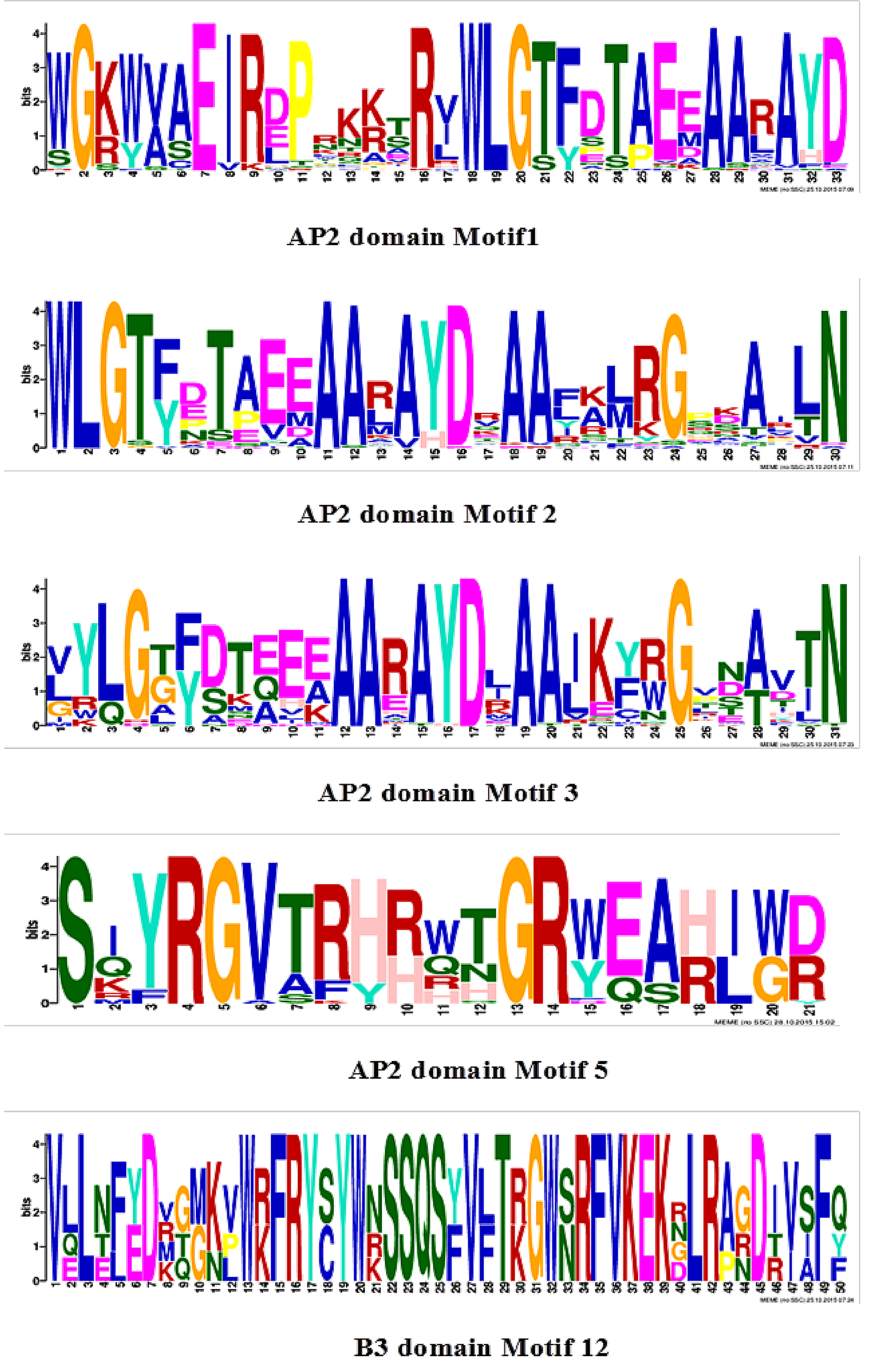

Supplement: Additional file 6: — Sequence Logo of the 5 motifs corresponding to AP2/ERF domains. (TIF 7418 kb) [file 12870_2016_859_MOESM6_ESM.tif]

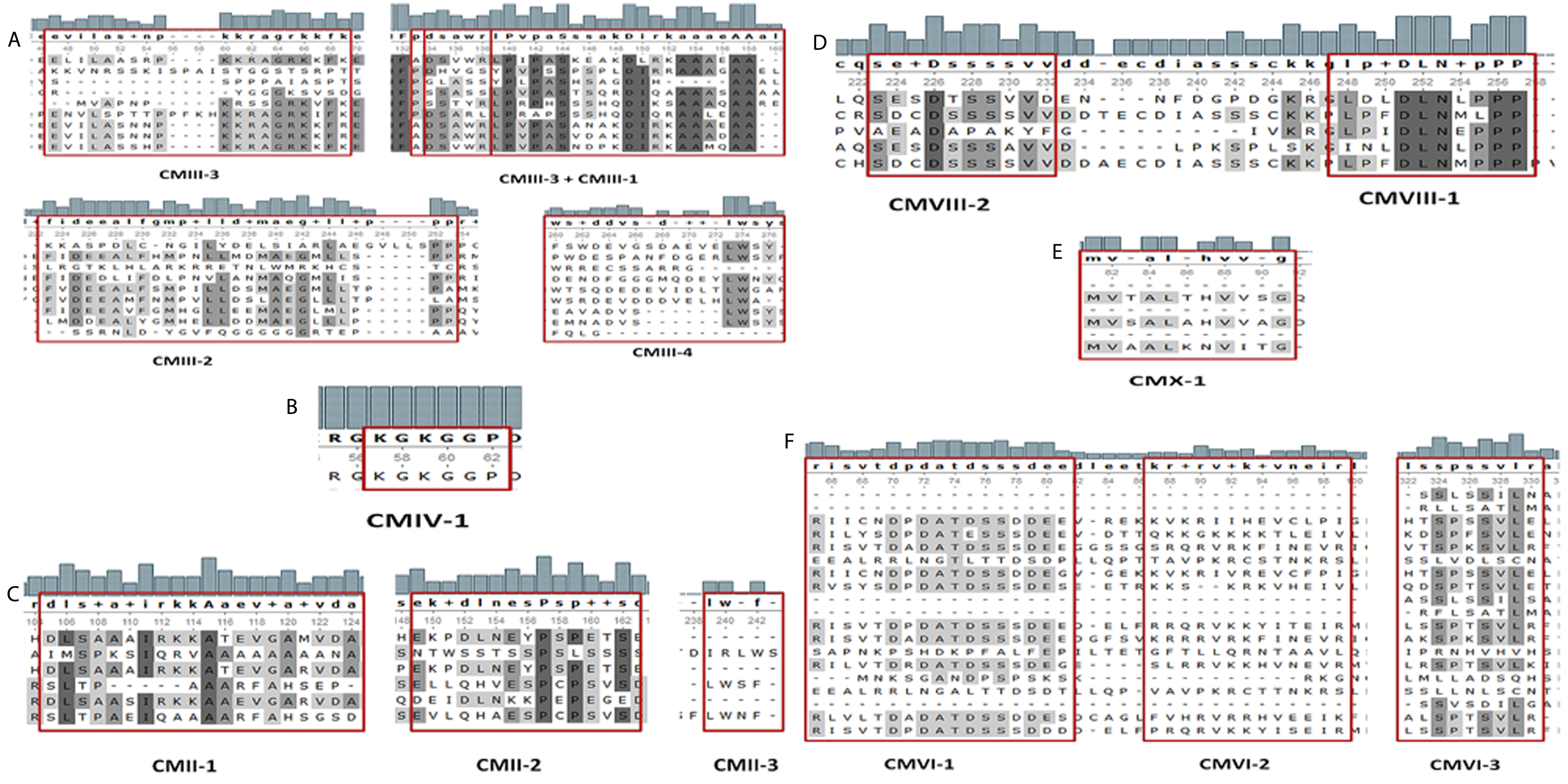

Supplement: Additional file 7: — Identification of conserved motifs in some sesame ERF functional groups as described by Nakano et al. (2006) in Rice and Arabidopsis. A: DREB1. B: DREB3. C: DREB5. D: ERF1. E: ERF4. F: ERF5. (TIF 15878 kb) [file 12870_2016_859_MOESM7_ESM.tif]
