# Supplementary material for: Insight into the AP2/ERF transcription factor superfamily in sesame and expression profiling of DREB subfamily under drought stress
Source: BMC Plant Biol. 2016 Jul 30;16:171. doi: 10.1186/s12870-016-0859-4 (PMC4967514; doi:10.1186/s12870-016-0859-4)
Supplement: Additional file 3: — Orthologous gene pairs of AP2/ERF and their localization in sesame, Arabidopsis, grape and tomato genomes. (DOCX 23 kb) [file 12870_2016_859_MOESM3_ESM.docx]

| Table Orthologous gene pairs of AP2/ERF and their localization in sesame, *Arabidopsis*, grape and tomato genomes | | | | | | | |
| --- | --- | --- | --- | --- | --- | --- | --- |
| Sesame genes | **LG** | **Start (bp)** | **End (bp)** | ***Tomato genes** | **Chr** | **Start (bp)** | **End (bp)** |
| AP2si5 | LG1 | 12752668 | 12756424 | XP_004235883.1 | 3 | 66774901 | 66779448 |
| AP2si27 | LG2 | 12873574 | 12875476 | XP_010325689.1 | 8 | 60355571 | 60357675 |
| AP2si28 | LG2 | 10206190 | 10208974 | XP_010316038.1 | 2 | 53258507 | 53262404 |
| AP2si29 | LG2 | 8573333 | 8575632 | XP_004252087.1 | 12 | 3501040 | 3504574 |
| AP2si61 | LG6 | 3504973 | 3507858 | XP_004250865.1 | 11 | 48379838 | 48384121 |
| AP2si78 | LG8 | 3650485 | 3651698 | XP_010325689.1 | 8 | 60355571 | 60357675 |
| AP2si118 | LG13 | 3857067 | 3860994 | XP_004250153.2 | 11 | 3758605 | 3764485 |
| AP2si121 | LG15 | 2746366 | 2749077 | NP_001233886.1 | 2 | 54077666 | 54080793 |
| AP2si125 | LG15 | 4360437 | 4363320 | XP_004231948.1 | 2 | 53258507 | 53262404 |
| AP2si3 | LG1 | 12265096 | 12265773 | XP_004235965.1 | 3 | 65952046 | 65953029 |
| AP2si11 | LG1 | 16569082 | 16569942 | XP_004229764.1 | 1 | 84228312 | 84229455 |
| AP2si12 | LG1 | 17411767 | 17412585 | XP_004231173.1 | 1 | 85341250 | 85342145 |
| AP2si13 | LG1 | 6369803 | 6370366 | XP_010317563.1 | 3 | 3711509 | 3715514 |
| AP2si64 | LG6 | 8886431 | 8887162 | XP_004245652.1 | 8 | 63607344 | 63609014 |
| AP2si30 | LG3 | 8228705 | 8229352 | XP_004241746.1 | 6 | 41797475 | 41798494 |
| AP2si17 | LG1 | 1637576 | 1638307 | XP_004245167.1 | 8 | 55477076 | 55477878 |
| AP2si18 | LG2 | 7533605 | 7534054 | XP_004238136.1 | 4 | 63332139 | 63332875 |
| AP2si36 | LG3 | 9547016 | 9549187 | NP_001234462.2 | 3 | 70333095 | 70335526 |
| AP2si33 | LG3 | 21866042 | 21866509 | XP_004238136.1 | 4 | 63332139 | 63332875 |
| AP2si50 | LG4 | 10290283 | 10290960 | XP_010317563.1 | 3 | 3711509 | 3715514 |
| AP2si51 | LG4 | 10436000 | 10436929 | XP_004236415.1 | 3 | 68370754 | 68371776 |
| AP2si54 | LG4 | 11416946 | 11417629 | XP_004239983.1 | 5 | 62397061 | 62398089 |
| AP2si54 | LG4 | 11416946 | 11417629 | XP_004235186.2 | 3 | 55030037 | 55031156 |
| AP2si79 | LG8 | 4878923 | 4879396 | XP_004250546.1 | 11 | 4719847 | 4720759 |
| AP2si80 | LG8 | 4868513 | 4869013 | XP_004250229.1 | 11 | 4727597 | 4728410 |
| AP2si81 | LG8 | 4848380 | 4848820 | XP_004250546.1 | 11 | 4719847 | 4720759 |
| AP2si91 | LG9 | 1391372 | 1393747 | XP_004247071.1 | 9 | 54598806 | 54604814 |
| AP2si96 | LG9 | 4156301 | 4158698 | XP_004247071.1 | 9 | 54598806 | 54604814 |
| AP2si98 | LG10 | 2427146 | 2427538 | XP_004234038.2 | 3 | 381291 | 384300 |
| AP2si99 | LG10 | 2436770 | 2437174 | XP_004247925.1 | 9 | 69522423 | 69522884 |
| AP2si101 | LG10 | 1253979 | 1255007 | XP_004229302.1 | 1 | 76297156 | 76298263 |
| AP2si106 | LG11 | 2989064 | 2989822 | XP_004229764.1 | 1 | 84228312 | 84229455 |
| AP2si107 | LG11 | 11923766 | 11924332 | XP_004249787.1 | 10 | 63342362 | 63342955 |
| AP2si110 | LG12 | 4413751 | 4414203 | XP_004232911.1 | 2 | 42687423 | 42688210 |
| AP2si114 | LG12 | 1044871 | 1045578 | NP_001239044.1 | 7 | 66870751 | 66871809 |
| AP2si120 | LG14 | 1022751 | 1023437 | NP_001239044.1 | 7 | 66870751 | 66871809 |
| AP2si6 | LG1 | 10257468 | 10258532 | XP_004238962.1 | 5 | 3986842 | 3988405 |
| AP2si24 | LG2 | 16330561 | 16331685 | XP_004238962.1 | 5 | 3986842 | 3988405 |
|  |  |  |  |  |  |  |  |
| Sesame genes | **LG** | **Start (bp)** | **End (bp)** | ****Arabidopsis* genes** | **Chr** | **Start (bp)** | **End (bp)** |
| AP2si27 | LG2 | 12873574 | 12875476 | NP_001030857.1 | 3 | 20114684 | 20118639 |
| AP2si29 | LG2 | 8573333 | 8575632 | NP_563990.1 | 1 | 5508507 | 5511735 |
| AP2si55 | LG5 | 509592 | 512524 | NP_195410.1 | 4 | 17400610 | 17403469 |
| AP2si61 | LG6 | 3504973 | 3507858 | NP_175530.2 | 1 | 18977284 | 18980618 |
| AP2si78 | LG8 | 3650485 | 3651698 | NP_001030857.1 | 3 | 20114684 | 20118639 |
| AP2si123 | LG15 | 751434 | 753203 | NP_001030857.1 | 3 | 20114684 | 20118639 |
| AP2si3 | LG1 | 12265096 | 12265773 | NP_172988.1 | 1 | 5283538 | 5284673 |
| AP2si4 | LG1 | 12505872 | 12506495 | NP_197901.1 | 5 | 8706790 | 8707748 |
| AP2si75 | LG7 | 10107809 | 10108408 | NP_197346.1 | 5 | 6116097 | 6117020 |
| AP2si13 | LG1 | 6369803 | 6370366 | NP_172721.1 | 1 | 4289944 | 4291017 |
| AP2si13 | LG1 | 6369803 | 6370366 | NP_176491.1 | 1 | 23367407 | 23368410 |
| AP2si13 | LG1 | 6369803 | 6370366 | NP_200012.1 | 5 | 21117113 | 21117787 |
| AP2si68 | LG6 | 11349354 | 11350031 | NP_182021.1 | 2 | 18537257 | 18538418 |
| AP2si14 | LG1 | 6407156 | 6407824 | NP_200015.1 | 5 | 21123958 | 21124948 |
| AP2si17 | LG1 | 1637576 | 1638307 | NP_196720.1 | 5 | 3727534 | 3728726 |
| AP2si18 | LG2 | 7533605 | 7534054 | NP_179915.1 | 2 | 9937998 | 9938875 |
| AP2si38 | LG3 | 14430110 | 14430820 | NP_172988.1 | 1 | 5283538 | 5284673 |
| AP2si33 | LG3 | 21866042 | 21866509 | NP_201520.1 | 5 | 26809135 | 26809689 |
| AP2si50 | LG4 | 10290283 | 10290960 | NP_200012.1 | 5 | 21117113 | 21117787 |
| AP2si96 | LG9 | 4156301 | 4158698 | NP_001078381.1 | 4 | 7613316 | 7615386 |
| AP2si107 | LG11 | 11923766 | 11924332 | NP_181186.1 | 2 | 15294303 | 15294857 |
| AP2si6 | LG1 | 10257468 | 10258532 | NP_173927.1 | 1 | 8981677 | 8983041 |
| AP2si6 | LG1 | 10257468 | 10258532 | NP_172784.1 | 1 | 4542168 | 4543742 |
| AP2si24 | LG2 | 16330561 | 16331685 | NP_172784.1 | 1 | 4542168 | 4543742 |
|  |  |  |  |  |  |  |  |
| Sesame genes | **LG** | **Start (bp)** | **End (bp)** | ***Grape genes** | **LG** | **Start (bp)** | **End (bp)** |
| AP2si5 | LG1 | 12752668 | 12756424 | XP_010656633.1 | 11 | 9496324 | 9499476 |
| AP2si5 | LG1 | 12752668 | 12756424 | XP_002272159.1 | 9 | 18937662 | 18941313 |
| AP2si27 | LG2 | 12873574 | 12875476 | XP_010661026.1 | 14 | 28882582 | 28886158 |
| AP2si27 | LG2 | 12873574 | 12875476 | XP_002270149.1 | 1 | 10859871 | 10861997 |
| AP2si28 | LG2 | 10206190 | 10208974 | AP2 | 11 | 18415754 | 18418676 |
| AP2si29 | LG2 | 8573333 | 8575632 | AP2 | 11 | 9496324 | 9499476 |
| AP2si61 | LG6 | 3504973 | 3507858 | XP_002285539.2 | 6 | 2228614 | 2233258 |
| AP2si78 | LG8 | 3650485 | 3651698 | XP_010661026.1 | 14 | 28882582 | 28886158 |
| AP2si121 | LG15 | 2746366 | 2749077 | NP_001267881.1 | 7 | 16436827 | 16440556 |
| AP2si123 | LG15 | 751434 | 753203 | XP_010661026.1 | 14 | 28882582 | 28886158 |
| AP2si125 | LG15 | 4360437 | 4363320 | XP_010645298.1 | 7 | 8883 | 13228 |
| AP2si117 | LG13 | 3030645 | 3031229 | XP_010652782.1 | 7 | 16436827 | 16440556 |

*Genebank Gene ID
