# Supplementary material for: Insight into the AP2/ERF transcription factor superfamily in sesame and expression profiling of DREB subfamily under drought stress
Source: BMC Plant Biol. 2016 Jul 30;16:171. doi: 10.1186/s12870-016-0859-4 (PMC4967514; doi:10.1186/s12870-016-0859-4)
Supplement: Additional file 5: — Motif sequences identified using MEME tools in sesame AP2/ERF genes. (DOCX 12 kb) [file 12870_2016_859_MOESM5_ESM.docx]

| Motif | Width | Best possible match |
| --- | --- | --- |
| 1 | 33 | WGKWVAEIRDPRKKTRIWLGTFDTAEEAARAYD |
| 2 | 30 | WLGTYDTAEEAARAYDRAAFKMRGPKAILN |
| 3 | 31 | VYLGTYDTQEEAARAYDIAAIKYWGVNAVTN |
| 4 | 15 | GVRQRPWGKWVAEIR |
| 5 | 21 | SIYRGVTRHHWTGRWEAHIWD |
| 6 | 29 | FPISDYEKEMEQMKNMTKQEYVHILRRQS |
| 7 | 20 | VAAICLRGPKAKLNFPHPVH |
| 8 | 11 | HKKYRGVRQRH |
| 9 | 24 | RLPRPASCSPKDIQAAAAKAAEMM |
| 10 | 29 | KRGSSKYRGVTLHKCGRWEAHMGQFHGKK |
| 11 | 29 | MRTVRIICTDPDATDSSSDEEDCLKKVKR |
| 12 | 50 | VLLNFYDMGMKVWKFRYCYWNSSQSYVFTKGWNRFVKEKNLRAGDIVIFY |
| 13 | 21 | IDEEEVFVMEDLLMDMLEGLL |
| 14 | 20 | FDISRYDVKRIMESNTLIIG |
| 15 | 15 | EPVPRKSIDTFGQRT |

**Table Motif sequences identified using MEME tools in sesame AP2/ERF genes**
