# Supplementary material for: Insight into the AP2/ERF transcription factor superfamily in sesame and expression profiling of DREB subfamily under drought stress
Source: BMC Plant Biol. 2016 Jul 30;16:171. doi: 10.1186/s12870-016-0859-4 (PMC4967514; doi:10.1186/s12870-016-0859-4)
Supplement: Additional file 8: — Multiple alignments of AP2/ERF protein sequences. (PDF 182 kb) [file 12870_2016_859_MOESM8_ESM.pdf]

Additional file 7 Multiple Alignment of sesame AP2/ERF protein sequences

```
AP2si1     1 Y--RGIRQR-PWGKWAAEIRDP--------RKGVR----------VWLGTFNTAEEAAKAYDTAARKIRG-
AP2si2     1 PVYRGVRMR-SWGKWVCEIRQPR-------MN-SR----------IWLGTYSTAEMAARAHDSAALSIKG-
AP2si3     1 K-YRGVRQR-HWGSWVSEIRHPL-------LK-RR----------VWLGTFDTAEEAARAYDEAA------
AP2si4     1 R-YRGVRQR-HWGSWVSEIRHPL-------LK-TR----------IWLGTFETAEDAAKAYDEAARIMC--
AP2si5     1 SKYRGVARHHHNGRWEARIGR---------VFGNKY---------LYLGTYATQEEAATAYDMAAIEYRG-
AP2si6     1 SRFKGVVPQ-PNGRWGAQIYEKH-------QR-------------VWLGTFNEEEEAARAYDTAAQRFRG-
AP2si7     1 K-YRGVRQR-KWGKWAAEIRDP--------IQHKR----------VWLGTYNTAEEASRAYELKRLEFEA-
AP2si8     1 K-FVGVRRR-KSGKYASEIRDP--------IIKKR----------VWLGTFATPEQASRAYLAKKKEID--
AP2si9     1 R-YRGVRRR-PWGTFAAEMRDPA-------KKGSR----------IWLGTYETPEEAAVAYDRAAFKLRG-
AP2si10    1 R-YRGVRRR-PWGKFAAEIRDPV-------KKGSR----------MWLGTYETPEEAALAYDRAAFKLRG-
AP2si11    1 R-YRGVRRR-PWGKFAAEIRDPV-------KKGSR----------MWLGTYETPEEAALAYDKAAFKLRG-
AP2si12    1 T-YRGVRKR-NWGRWVCEIREPR-------KK-SR----------IWLGTYPTAEMAARAHDVAALAIKG-
AP2si13    1 R-FVGVRQR-PSGRWVAEIKDSS-------QR-VR----------LWLGTYDTPEEAARAYDEAARALRGE
AP2si14    1 PVYRGVRRR-NSDKWVCELREPS-------KQ-KR----------IWLGGVPTAEMAARAHDVAALALRG-
AP2si15    1 PIYRGIRSR--SGKWVSEIREPR-------KT-TR----------IWLGTYPSPEMAAAAYDVAALALKG-
AP2si16    1 K-FRGVRQR-PWGKWAAEIRDPL-------RR-VR----------LWLGTYDTAEEAAMVYDHAAIQLRG-
AP2si17    1 Y--RGVRQR-HWGKWVAEIRLPQ-------NR-IR----------VWLGTYETAEAAAYAYDRAAYKLRGE
AP2si18    1 PVYRGVRMR-NWGKWVSEIREPR-------KK-SR----------IWLGTFPTPEMAARAHDVAALSIKG-
AP2si19    1 Y--RGIRMR-KWGKWVAEIREPN-------KR-SR----------IWLGSYSSPVAAARAYDTAVFYLRG-
AP2si20    1 K-YKGVRMR-SWGSWVSEIRAPN-------QK-TR----------IWLGSYSTPEAAARAYDAALLCLKG-
AP2si21    1 K-YRGVRQR-PWGKWAAEIRDP--------HKAAR----------VWLGTFDTAEAAARAYDEAALRFRG-
AP2si22    1 Y--RGVRQR-HWGKWVAEIRLPK-------NR-TR----------LWLGTFDTAEEAALAYDKAAYQLRG-
AP2si23    1 SKYIGVRKR-KYGRWVSEIREPG-------KK-TR----------IWLGSFDTPEMAAAAYDAAALQLRG-
AP2si24    1 R-YRGVRRR-PWGRYAAEIRDPQ-------TK-ER----------RWLGTFDTAEEAACAYDCAARAMRG-
AP2si25    1 SRFKGVVPQ-PNGRWGAQIYEKH-------QR-------------VWLGTFNEEEEAARAYDTAAQRFRG-
AP2si26    1 K-YRGVRQR-KWGKWAAEIRDP--------IKHKR----------VWLGTYNTAEEASRAYELKRLEFEA-
AP2si27    1 TKFVGVRRR-NWGKYSAEIRDP--------FAKKR----------VWLGTFSTAEEASRAYLSKK------
AP2si28    1 SRFRGVSRHRWTGRYEAHLWDKGSWNVTQKKKGKQ-------------GAYDEEEAARAYDLAAIKYWG-
AP2si29    1 I-FRGVTRHRWTGRYEAHLWDNSCRREGQSRKGRQ-------------GGYDKEEKAARAYDLAALKYWG-
AP2si30    1 SIYRGVTRHRWTGRYEAHLWDKNCWNEAQNKKGRQ-------------GAYDDEEAAAHAYDLAALKYWG-
AP2si31    1 PVYRGVRMR-SWGKWVSEIRHPR-------KK-SR----------IWLGTYPTAEMAARAHDVAALSIKG-
AP2si32    1 SQYRGVTRHRWTGRYEAHLWDNSCRKEGQTRKGRQV----------YLGGYDKEEKAARAYDLAALKYWG-
AP2si33    1 SQYRGVTRHRWTGRYEAHLWDNSCKKEGQTRKGRQV----------YLGGYDMEEKAARAYDLAALKYWG-
AP2si34    1 Y--RGIRMR-KWGKWVAEIREPN-------KR-SR----------IWLGSYSSPVAAARAYDTALFYLRG-
AP2si35    1 SQYRGVTRHRWTGRYEAHLWDNSCKKEGQSRKGRQ-------------GGYDMEEKAARAYDLAALKYWG-
AP2si36    1 Y--RGVRQR-HWGKWVAEIRLPK-------NR-TR----------LWLGTFDTAEEAALAYDKAAYKLRG-
AP2si37    1 Y--RGIRQR-PWGKWAAEIRDP--------RKGVR----------VWLGTFNTAEEAARAYDTEARRIRG-
AP2si38    1 Y--RGVRRR-PWGKFAAEIRDST-------RNGIR----------VWLGTFDTAEAAALAYDQAALSTRG-
AP2si39    1 K-FRGVRQR-HWGSWVSEIRHPL-------LK-RR----------VWLGTFETAEEAARAYDEAA------
AP2si40    1 S-YRGVRKR-PWGRWSAEIRDRI-------GR-CR----------HWLGTFDTAEEAARAYDAAARRLRG-
AP2si41    1 R-YRGVRQR-HWGSWVSEIRHPL-------LK-TR----------IWLGTFETAEDAAKAYDEAARIMC--
AP2si42    1 H-YRGVRQR-PWGKWAAEIRDP--------KKAAR----------VWLGTFDSAEAAALAYDEAALRFKG-
AP2si43    1 Y--RGVRRR-PWGKYAAEIRDST-------RNSVR----------VWLGTFDTAEEAALAYDQAAFAMRG-
AP2si44    1 SIYRGVTRHRWTGRYEAHLWDNSCRREGQTRKGRQV----------YLGGYDKEEKAARAYDLAALKYWG-
AP2si45    1 CEYRGVRQR-TWGKWVAEIREPN-------KR-TR----------LWLGSFATAEEAAMAYDEAARRLYG-
AP2si46    1 SQYRGVTFYRRTGRWESHIWD----------CGKQ----------VYLGGFDTAHAAARAYDRAAIKFRG-
AP2si47    1 CNYRGVRQR-TWGKWVAEIREPH-------RG-SR----------LWLGTFGTALEAALAYDEAAKAMYG-
AP2si48    1 R-YRGVRQR-SWGKWVAEIREPR-------KR-TR----------RWLGTFSTAEDAARAYDRAAI-----
AP2si49    1 K-FRGVRQR-PWGKWAAEIRDPS-------RR-VR----------LWLGTYDTAEEAAMVYDNAAIKLRG-
AP2si50    1 PVYRGIRCR--SGKWVSEIREPR-------KT-TR----------VWLGTYPTPEMAAAAYDVAALALKG-
AP2si51    1 PVYRGVRRR-NSGKWVCEVREPN-------KK-SR----------IWLGTFPTVEMAARAHDVAAMALRG-
AP2si52    1 K-FVGVRQR-PSGRWVAEIKDSL-------QK-VR----------LWLGTFDTAEDAARAYDNAARQLRG-
AP2si53    1 R-YRGVRRR-PWGKYAAEIRDPI-------RKGSR----------VWLGTYDTAVDAARAYDCAAFKMRG-
AP2si54    1 H-YRGVRQR-PWGKFAAEIRDPN-------RKGTR----------VWLGTFDTAVEAARAYDRAAFKLRG-
AP2si55    1 H-YRGVRRR-PWGKFAAEIRDPA-------KNGAR----------VWLGTYETAEEAALAYDRAAYKMRG-
AP2si56    1 SQYRGVTFYRRTGRWESHIWD----------CGKQ----------VYLGGFDTAHAAARAYDRAAIKFRG-
AP2si57    1 CKYKGVRKR-KWGKYVSEIRLPN-------SR-AR----------IWLGSYDTAEKAARAFDAALFCLRG-
AP2si58    1 V-YRGVRMR-AWGKWVSEIREPR-------KK-SR----------IWLGTFATPEMAARAHDVAALAIKG-
AP2si59    1 SKYRGVARHHHNGRWEARIGR---------VCGNKY---------LYLGTYSTQEEAAAAYDMAAIEFRG-
AP2si60    1 R-FRGVRRR-PWGKFAAEIRDSN-------NHGAR----------VWLGTFATAEEAARAYDRAAYTMRG-
AP2si61    1 K-YRGIRRR-PWGKFAAEIRDPN-------RNGAR----------LWLGTFETAEEAARAYDRAAYALRGH
AP2si62    1 SIYRGVTRHRWTGRYEAHLWDNSCRREGQSRKGRQV----------YLGGYDKEEKAARAYDLAALKYWG-
AP2si63    1 Y--RGIRQR-PWGKWAAEIRDP--------RKGVR----------VWLGTYNTAEEAARAYDREARKIRG-
AP2si64    1 PQFRGVRKR-PWGRYAAEIRDPW-------KK-TR----------KWLGTFDTAEEAALAYDEAARNLRG-
AP2si65    1 K-FRGVRQR-PWGKWAAEIRDPL-------RR-VR----------LWLGTYDTAEEAAMVYDHAAIQLRG-
AP2si66    1 L-YRGIRSR--SGKWVSEIREPK-------KT-TR----------IWLGTYPSPEMAAAAYDVAALALKG-
AP2si67    1 PVYRGVRRR-NSNKWVCELREPS-------KQ-KR----------IWLGSYPTPEMAARAHDVAALALRG-
```

```
AP2si68    1 R-YRGVRRR-PWGKYAAEIRDPA-------RRGSR-----------VWLGTFDTAVEAAKAYDRAAFKMRG-
AP2si69    1 H-YRGVRQR-PWGKFAAEIRDPA-------KNGAR-----------VWLGTYETAEDAALAYDRAAYRMRG-
AP2si70    1 F--RGVRRR-SWGKWVSEIREPR-------KK-SR-----------IWLGTFATAEMAARAHDAAAIAIKG-
AP2si71    1 K-YKGVRKR-KWGKYVSEIRLPN-------SR-ER-----------IWLGSYDTAEKAARAFDAALFCLRG-
AP2si72    1 K-FRGVRQR-QWGSWVSEIRHPL-------LK-KR-----------IWLGTFETAEAAARAYDEAAILM---
AP2si73    1 K-FRGVRQR-PWGRWAAEIRDPT-------RG-KR-----------VWLGTYDTPEEAAAVYDRAAVKLKG-
AP2si74    1 SKFKGVVSQ-QNGHWGAQIYANH-------QR--------------VWLGTFKSEVEAAMAYDSAAIKLR--
AP2si75    1 CNYRGVRQR-TWGKWVSEIREPH-------RG-SR-----------LWLGTFKTANEAALAYDEAARAMYGH
AP2si76    1 K-FRGVRQR-PWGKWAAEIRDPS-------RR-VR-----------LWLGTYDTAEEAAMVYDNAAIKLRG-
AP2si77    1 SIYRGVTRHRWTGRYEAHLWDNSCRREGQTRKGRQ--------------GGYDKEEKAARAYDLAALKYWG-
AP2si78    1 T-YRGVRQR-TWGKWVAEIREPN-------RG-AR-----------VWLGTFNTSLEAAQAYDDAARRLYG-
AP2si79    1 T-YKGVRQR-TWGKWVAEIREPN-------RG-SR-----------VWLGTFDTSYDAAVAYDTAARKLYG-
AP2si80    1 R-FVGVRQR-PSGRWVAEIKDTI-------QK-IR-----------VWLGTFDTAEEAARAYDEAACLLRG-
AP2si81    1 SRFRGASRHRWTGRDEAHLWDKQSWNATQKKKGKQ--------------GAYDNDEAAARAYDLAALKYWG-
AP2si82    1 R-YIGVRRR-PWGKYAAEIRDST-------RNGKR-----------VWLGTFDTEEEAALAYDQAAFATRG-
AP2si83    1 H-YIGVRKR-PWGKYAAEIRDST-------RNGRR-----------VWLGTFNTEEEAALAYDQAAFAMRG-
AP2si84    1 R-YIGVRKR-PWGKYAAEIRDST-------RNGKR-----------VWLGTFNTEEEAALAYDQAAFATRG-
AP2si85    1 N-YRGVRQR-PWGKWAAEIRDP--------RKAAR-----------VWLGTFETAEDAARAYDRAAIEFRG-
AP2si86    1 Y--RGVRQR-HWGKWVAEIRLPR-------NR-TR-----------LWLGTFDTAEEAALAYDREAFKLRGE
AP2si87    1 G----VRRR-PWGRYAAEIRDPS-------TK-ER-----------HWLGTFDTAEEAALAYDRAARSMRG-
AP2si88    1 G----VRRR-PWGRYAAEIRDPL-------TK-ER-----------HWLGTFDTAEEAALAYDRAARSMRG-
AP2si89    1 H-YRGVRQR-PWGKWAAEIRDP--------KKAAR-----------VWLGTFDTAEDAALAYDEAALRFKG-
AP2si90    1 F--KGVVPQ-QNGHWGAQIYANH-------HR--------------VWLGTFKSEIDAAMAYDSAAIKLR--
AP2si91    1 R-YRGVRRR-PWGRYAAEIRDPQ-------SK-ER-----------RWLGTFDTAEEAACAYDCAARAMRG-
AP2si92    1 Y--RGVRQR-HWGKWVAEIRLPK-------NR-TR-----------LWLGTFDTAEEAALAYDKAAYKLRGE
AP2si93    1 PVYKGVRRK-S------EVREPN-------KK-SR-----------IWLGTFPDPETAAIAHDVAALALRGE
AP2si94    1 CVMRGVYFK--NLKWQAAIKVDK-------KQ--------------IHLGTVGSQEEAARLYDRAAFM----
AP2si95    1 K-FVGVRQR-PSGKWVAEIKNTT-------QK-IR-----------MWLGTFDTAEEAAQAYDEAACLLRG-
AP2si96    1 R-YRGVRQR-PWGKWAAEIRDP--------YKAAR-----------VWLGTFDTAEEAARAYDEAALRFRG-
AP2si97    1 PVYRGVRRR-SRGKWVCEVREPN-------KK-SK-----------IWLGTFSNPEMAAVAHDVAALALRG-
AP2si98    1 C-----------GK-------------------Q------------VYLGGFDTALAAARAYDRAAIKFRG-
AP2si99    1 M--RGVYYK--NRKWQAAIKVDK-------KQ--------------IHLGTVGSEEEAARLYDRAAFM----
AP2si100   1 K-YRGVAKK-----WEESQR---------------R-------------WRCEWD------------------
AP2si101   1 C-----------GK-------------------Q------------VYLGGFDTALAAARAYDRAAIKFRG-
AP2si102   1 K-YRGVRKR-PWGKYAAEIRDSA-------RQGAR-----------VWLGTFNTAEEAARAYDKAAYAMRGH
AP2si103   1 R-YRGVRQR-PWGKFAAEIRDPT-------RQGAR-----------LWLGTFDTAEEAARAYDKAAFKLRGH
AP2si104   1 F--RGVRKR-PWGKFAAEIRDST-------RNGVR-----------VWLGTFDTAEAAAMAYDQAAFAMRG-
AP2si105   1 G----VRRR-PWGRYAAEIRDPT-------TK-ER-----------HWLGTFDTAQEAALAYDRAALSIKG-
AP2si106   1 SIYRGVTRHRWTGRFEAHLWDKTTWNSIQNKRGRQ--------------RAYDNEEDAARTYDLAALKYWG-
AP2si107   1 F--RGVRQR-HWGKWVAEIRLPR-------NR-TR-----------VWLGTFDTAEEAAFAYDTAAYILR--
AP2si108   1 N-YRGVRQR-PWGKWAAEIRDP--------RRAQR-----------VWLGTFETAEDAARAYDRAAIEFRG-
AP2si109   1 K-FRGVRQR-PWGKWAAEIRDPT-------RR-TR-----------VWLGTYDTAEEAATAYDRAAIQIRG-
AP2si110   1 Y--RGVRRR-SWGKWVCEIREPR-------KK-SR-----------IWLGTYPTAEMAARAHDVAALAIKGE
AP2si111   1 Y--RGVRRRKSSGKWVSEIREPR-------TP-NR-----------IWLGTFPTPEMAAVAYDVAAFALKG-
AP2si112   1 CKYKGVRKR-KWGKYVSEIRLPN-------CR-ER-----------IWLGSYDTAEKAARAFDAALFCLRG-
AP2si113   1 PVYRGVRMR-AWGKWVTEIREPR-------KK-SR-----------IWLGTFATPEMAARAHDVAALAIKG-
AP2si114   1 H-YRGVRKR-PWGRYAAEIRDPW-------KK-TR-----------VWLGTFDTPEEAALAYDGAARSLRG-
AP2si115   1 H-FRGVRKR-PWGRYAAEIRDPG-------KK-SR-----------VWLGTFDTAEEAARAYDNAARQFRG-
AP2si116   1 N-YRGVRRR-PWGKFAAEIRDSN-------RQGAR-----------IWLGTFETAEEAALAYDKAAFRMRG-
AP2si117   1 H-YRGVRRR-PWGKYAAEIRDST-------RKGAR-----------IWLGTFETAEEAAMAYDKAALRIRG-
AP2si118   1 R-FRGVRKR-PWGRFAAEIRDPW-------KK-TR-----------VWLGTFDSAEEAARAYDSAARSLRG-
AP2si119   1 Y--RGVRQR-HWGKWVAEIRLPR-------KR-TR-----------LWLGTFESAEKAALAYDRQAFRLRGE
AP2si120   1 Y--KGIRMR-KWGKWVAEIREPN-------KR-SR-----------IWLGSYST----AR------------
AP2si121   1 SQYRGVTFYRRTGRWESHIWY----------INTQ-----------IYV------------Y----I-----
AP2si122   1 SIYRGVTRHRWTGRYEAHLWDNSCRREGQARKGRQASTDPPTPTPVYLGGYDKEDKAARAYDLAALKYWG-
AP2si123   1 Y--RGVRQR-HWGKWVAEIRLPK-------NR-TR-----------LWLGTFDTAEDAALAYDKAAYKLRGE
AP2si124   1 R-FRGVRKR-PWGRYAAEIRDPW-------KK-TR-----------VWLGTFDSAEEAARAYDTAARSLRG-
AP2si125   1 SQYRGVTFYRRTGRWESHIWD----------CGKQ-----------VYLGGFDTAHAAARAYDRAAIKFRG-
AP2si126   1 Y--KGIRMR-KWGKWVAEIREPN-------KR------------------------------------------
AP2si127   1 SRFRGVSKHRWTGRYEAHLWDKLSWNVTQKKKGKQ--------------GAYDDEEAAARAYDLAALKYWG-
AP2si128   1 R-FVGVRQR-PSGRWVAEIKDTI-------QK-IR-----------VWLGTFDTAEEAARAYDEAACLLRG-
AP2si129   1 SQYRGVTRHRWTGRYEAHLWDNSCKKEGQSRKGRQV-----------YLGGYDMEEKAARAYDLAALKYWG-
AP2si130   1 H-YRGVRKR-PWGRYAAEIRDPG-------KK-SR-----------VWLGTFDTAEEAARAYDAAAREFRG-
AP2si131   1 H-YRGVRQR-PWGKFAAEIRDPA-------KNGAR-----------VWLGTYETAEEAALAYDKAAYRMRG-
AP2si132   1 H-YRGVRQR-PWGKFAAEIRDPT-------RKGTR-----------VWLGTFDTAVEAAKAYDRAAFKLRG-
```
